# Underestimated virus impaired cognition-more evidence and more work to do

**DOI:** 10.3389/fimmu.2025.1550179

**Published:** 2025-05-12

**Authors:** Maher Un Nisa Awan, Faisal Mahmood, Xiao-bin Peng, Fenshuang Zheng, Jun Xu

**Affiliations:** ^1^ Department of Neurology, The Affiliated Hospital of Yunnan University, Kunming, China; ^2^ Central Laboratory, Liver Disease Research Center and Department of Infectious Disease, The Affiliated Hospital of Yunnan University, Kunming, China; ^3^ School of Medicine, Yunnan University, Kunming, Yunnan, China; ^4^ Department of Emergency, The Affiliated hospital of Yunnan University, Kunming, China; ^5^ Department of Neurology, Beijing Tiantan Hospital, Capital Medical University, Beijing, China

**Keywords:** neurodegeneration, virus, antiviral drugs, impaired cognition, dementia

## Abstract

Neurodegenerative disorders (NDs) are chronic neurological diseases that can be of idiopathic, genetic, or potentially infectious origin. Although the exact cause of neurodegeneration is unknown, it might be result of a confluence of age, genetic susceptibility factors, and environmental stresses. The blood-brain barrier shields the brain from the majority of viral infections, however neurotropic viruses are able to breach this barrier and infect central nervous system. Growing research points to a possible connection between viruses and neurodegenerative diseases, indicating that virus-induced neuroinflammation and disruption of neuronal protein quality control may play a role in the initial stages of disease progression. The diagnosis and treatment of NDs are urgent and challenging. Even though there is limited clinical evidence to support the use of antiviral medications and their dose regimens within the central nervous system (CNS), with the exception of acyclovir, they are currently utilized to treat various viral CNS infections. Understanding the neuropathogenesis of viral CNS infection may help with targeted diagnosis and treatment plans by focusing on the molecular mechanisms of the CNS. It may also be helpful in the search for new antiviral drugs, which are crucial for better managing these neurotropic viral infections. This review focuses on new findings linking viral infection to NDs and explores how viral modifications of cellular functions can impact the development of neurodegeneration and will also explore the therapeutic potential of antiviral drugs in NDs.

## Introduction

Neurodegenerative diseases (NDs) are chronic degenerative disorders of the central nervous system (CNS) that are characterized by the chronic and progressive loss of the structure and function of neurons (1). Millions of people worldwide are impacted by them, making them the fourth most common cause of mortality in developed nations. Furthermore, their influence is growing in developing countries. With an increasing lifespan, it is expected that the incidence rate will rise. Even with extensive investigation, most NDs’ basic root causes are still poorly understood (1, 2). Numerous intracellular mechanisms, such as apoptosis, inefficient axonal transport, mitochondrial malfunction, and protein degradation, are linked to neurodegenerative diseases (3). The etiology of numerous neurodegenerative illnesses has also been linked to long-term viral infections, malnutrition, exposure to heavy metals in the environment, autoimmune reactions, vascular disorders, head trauma, brain fluid buildup, and alterations in neurotransmitter concentrations (2, 4, 5). Viral infections can infiltrate the immune system and other organ systems, resulting in a variety of symptoms (6).

The majority of NDs have a pathogenic connection to the accumulation and aggregation of cellular proteins (7, 8). Notably, dementia with Lewy bodies, multiple systems atrophy (MSA), and Parkinson’s disease (PD) have all been associated with α-synuclein (α-syn) aggregates (9). Alzheimer’s disease (AD) patients also have extracellular amyloid-β (Aβ) plaques and intraneuronal tangles of hyperphosphorylated tau in their brains (10). Like prions, these pathogenic proteins can aggregate and form pathogenic plaques, which leads to the eventual development of NDs (11, 12). A significant contributing component to these processes is an imbalance in the cellular mechanisms that control the creation of misfolded proteins and their breakdown, or protein homeostasis (13). The potential for viral infections to significantly disrupt protein homeostasis makes cells more vulnerable to protein misfolding (14). Moreover, maintaining protein homeostasis may benefit from the release of pro-inflammatory cytokines and chemokines in response to a virus (15). Up-regulation of pro-inflammatory cytokines plays a dual role in neurodegeneration and neuroprotection. Activated microglia can cause harm by releasing pro-inflammatory cytokines such IL-1β, IL-6, and TNF-α, which affect surrounding brain tissue.

Therefore, it is believed that viruses, particularly neurotropic viruses, play a part in the genesis of various NDs. **Table 1** lists the several viruses that are believed to be involved in NDs.

**Table 1:** Viruses in Neurodegeneration.

Neurodegenerative disorders (NDs)	Virus	References
Parkinson’s disease (PD)	Coxsackievirus B3 (CVB3)	(16, 17)
	Human Immunodeficiency Virus (HIV)	(18)
	Influenza A virus (IAV)	(19, 20)
	West Nile Virus (WNV)	(21, 22)
	Western equine virus (WEV)	(23, 24)
	Hepatitis C virus (HCV)	(25–28)
	Hepatitis B virus (HBV)	(28, 29)
	Japanese encephalitis virus (JEV)	(30, 31)
	Herpes simplex virus (HSV)	(32)
	Varicella-Zoster Virus (VZV)	(33)
	Epstein-Barr virus (EBV)	(34, 35)
Alzheimer’s disease (AD)	Herpes simplex virus (HSV)	(36, 37)
	Human immunodeficiency virus (HIV)	(18, 38, 39)
	Human Herpesvirus (HHV)	(40)
	Hepatitis B virus (HBV)	(41, 42)
	Hepatitis C virus (HCV)	(41–44)
	Epstein-Barr virus (EBV)	(35, 45)
	Varicella Zoster Virus (VZV)	(46, 47)
Amyotrophic lateral sclerosis (ALS)	Enteroviruses (EVs)	(48)
	Herpes simplex virus (HSV)	(49)
(Multiple sclerosis) MS	Epstein-Barr virus (EBV)	(50)
	HSV	(51)
Vascular dementia	VZV	(52)

## Viruses in neurodegeneration

**Figure 1:**
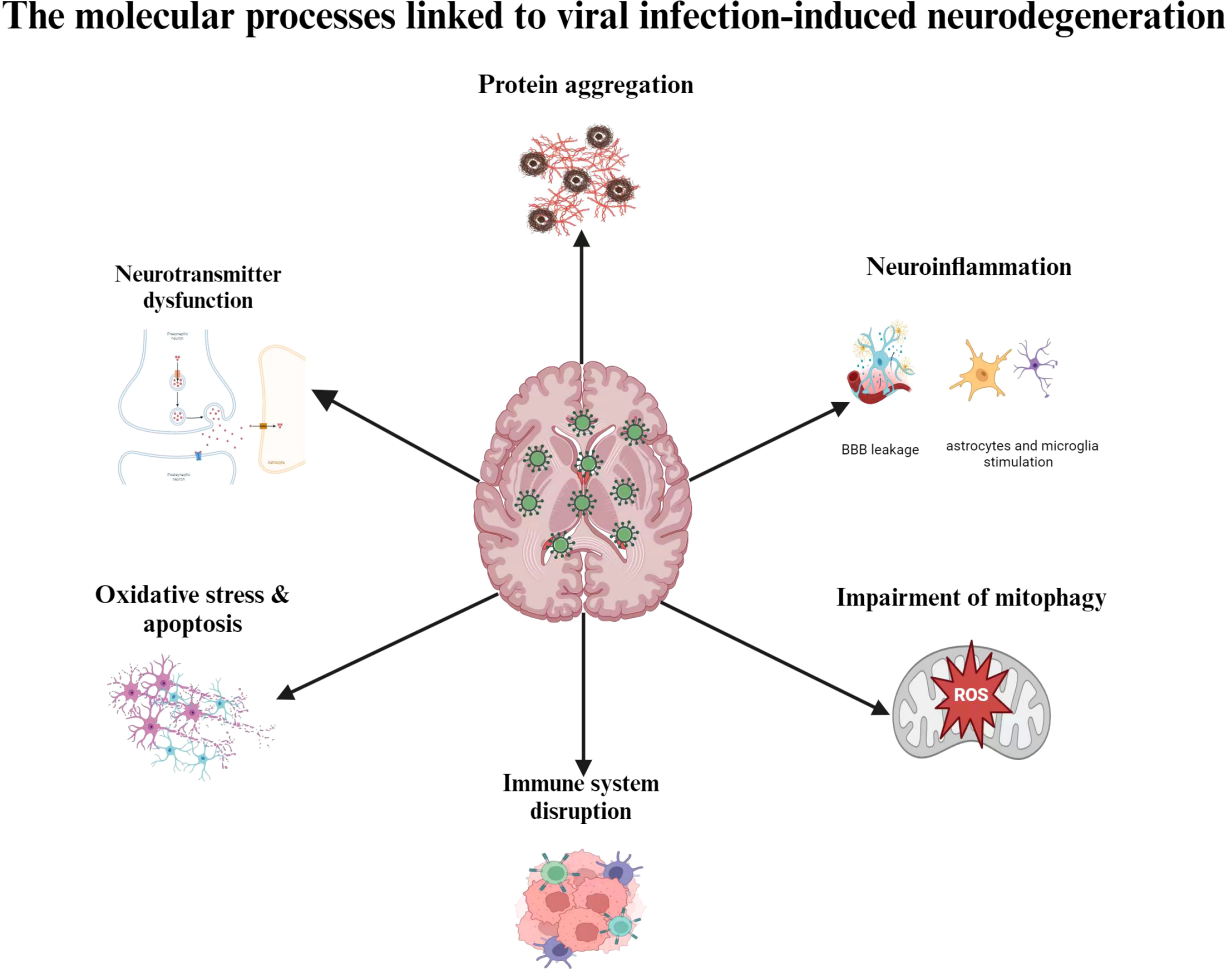
Molecular mechanisms adapted by viruses in causing Nds.

It is likely that aging, genetic vulnerability, and environmental stressors all play a part in this process, even if the precise etiological reasons of NDs are still not entirely understood. There is mounting evidence that suggests viral infections, especially neurotropic viruses, may play a factor in the onset and progression of depressions that are not diagnosed. The progressive loss of cognitive, motor, and behavioral abilities is a hallmark of neurodegenerative illnesses like AD, PD, and amyotrophic lateral sclerosis (ALS) (53). Despite early assumptions that neuroinflammation results from neurodegeneration, further studies have demonstrated that neuroinflammation can both cause and accelerate the development of NDs. The hypothesis that neuroinflammation causes neurodegeneration was reinforced by genome-wide association studies (GWAS) that identified immune-related genes, including as CD33 and TREM2, as risk factors for AD (54). Additionally, it has been suggested that neuroinflammatory processes are largely influenced by the ϵ4 allele of the apolipoprotein E gene (APOE ϵ4), which is the most powerful genetic risk factor for AD and accounts for around 10–20% of the risk of late-onset illness (55). These genetic factors increase the risk of NDs, but they are not sufficient to cause the condition on their own. There is increasing evidence that viruses and neurodegenerative illnesses are associated (56–59). Virus-induced neuroinflammation and disruption of neuronal protein quality control may also be involved in the early phases of illness development (60). Viruses can begin and/or aggravate degenerative processes because they have the capacity to take over the host cell’s internal machinery and induce inflammation. Viral infections can stimulate astrocytes and microglia or allow peripheral immune cells to invade the central nervous system, which can result in neuroinflammation (61). Certain viruses can disrupt neuronal activities, cause neuronal death, or trigger lytic egress from infected neurons, all of which can lead to neurodegeneration. Numerous negative outcomes are brought on by CNS viral infections, such as elevated morbidity and mortality as well as mild to severe neurological aftereffects, shown in **Figure 1**. Viral infections have a wide range of impact on neuronal dysfunction, including promoting chronic inflammation, inducing cellular oxidative stress, impairing mitophagy, interfering with mitochondrial dynamics, enhancing metabolic rewiring, altering neurotransmitter systems, and inducing misfolded and aggregated pathological proteins linked to neurodegenerative diseases. These pathogenetic processes cause a multifaceted brain injury that results in neuronal and brain dysfunctions. By interfering with the immune system, it can either directly or indirectly induce encephalitis (62). Neurotropic viral infections have an impact on a multitude of factors related to neuronal dysfunction. These include the induction of misfolded and aggregated pathological proteins linked to neurodegenerative diseases, the promotion of chronic inflammation, the induction of cellular oxidative stress, the impairment of mitophagy, the interaction with mitochondrial dynamics, the enhancement of metabolic rewiring, the modification of neurotransmitter systems (63). A complex brain injury brought on by these pathogenetic mechanisms gives rise to specific brain and neuronal dysfunction (64). Understanding the molecular mechanisms behind the neurophathogenesis associated with viral infection-induced neurodegeneration could lead to the development of efficient prophylactic, therapeutic, and preventive measures against CNS virus infections.

## Molecular mechanisms associated with viral infection-related neurodegeneration

Viruses can directly cause neuronal dysfunction through their cytolytic effects, and they can also indirectly cause neuronal degeneration through a variety of mechanisms, including the expression of viral genes that disrupt the host’s immune system and cellular functions, bystander inflammatory responses, or apoptosis (65). Herpes simplex virus (HSV; family Herpesviridae) and human immunodeficiency virus (HIV; family Retroviridae) are two examples of viruses that exhibit oxidative stress and cause latent or delayed infections. Microglia and brain cells were found to produce intracellular ROS in response to HSV-1 infection. In cultured mouse neural cells, HSV-1 infection results in oxidative stress and triggers the production of bioactive lipid peroxidation byproducts, MDA/hydroxyalkenals (HAEs), which are essential for viral replication (66). A number of HIV-1 component proteins, through various processes, increase the formation of ROS in neural cells, including neurons, microglial cells, and astrocytes. ROS generation and substantial DNA damage are induced by the HIV-1 transactivator of transcription (Tat) protein (67). Nitroxidative stress marker proteins, including cytochrome P450-2E1 (CYP2E1), iNOS, and NADPH oxidase, are found to be elevated in the brains of HIV-1 transgenic rats. Neuronal cell death in HIV-1 transgenic rats was linked to markedly increased hippocampal levels of activated caspase-3 and BCL2 associated X (BAX) in the HIV-1 model. In conjunction with the activation of MAPK pathways mediated by ERK and JNK and the reduction of B-cell lymphoma 2 (BCL-2) expression, HIV-1 gp120 protein causes death in neurons and microglial cells (68). In neurons and glial cells, JEV (family Flaviviridae) infection raises the concentrations of superoxide anions (O2.-), nitric oxide (NO), and peroxynitrite (OONO-) (69). Neuronal cells infected with other members of the Flaviviridae family, such as West Nile virus (WNV) (70) and dengue virus type 2 (DENV-2), also showed excessive O2.-production during viral infection (71, 72), which resulted in host cell apoptosis.

The coronavirus is the largest kind of RNA virus, human proteins that interact with SARS-CoV-2 proteins have also been implicated in a number of biological processes linked to aging and neurodegenerative diseases, including lipid metabolism, responses to oxidative stress, and problems with protein homeostasis and mitochondrial function (73). Due to immune-response dysregulation and the effect of COVID-19-related discomfort on cognitive performance, people with AD seem to be at a higher risk of experiencing severe COVID-19 outcomes. COVID-19-induced systemic inflammation may be a factor in neurodegeneration and cognitive impairment.PD patients have a higher case fatality rate during COVID-19 infections, but the underlying mechanisms remain unclear. Additional research is required to determine whether the diseases share any pathophysiological pathways or risk factors. Akinetic-rigid parkinsonism that develops after severe COVID-19 instances begs the question of how the virus affects dopamine pathways. Due to respiratory muscle involvement and heightened vulnerability to respiratory problems during the pandemic, ALS patients face challenges. In COVID-19 cases, some genetic variants associated with familial ALS, like C9orf72 repeat expansions, may affect the severity of the disease (74). A study revealed that Intranasal infection of C57BL/6J mice with the SARS-CoV-2 Beta strain causes Ly6Chi monocyte infiltration of the central nervous system and activation of microglia. SARS-CoV-2, but not H1N1 influenza virus, raises brain IL-1β levels and causes IL-1R1-mediated loss of hippocampus neurogenesis, resulting in post-acute cognitive impairments. Vaccination with a low dosage of adenoviral-vectored spike protein suppresses hippocampus synthesis of IL-1β during breakthrough SARS-CoV-2 infection, resulting in neurogenesis loss and memory impairments (75, 76).

Influenza virus, belonging to Orthomyxoviridae family, which are negative sense, single-stranded, segmented RNA viruses. Influenza A virus was found to be present in substantia nigra pars compacta (SNpc) from postmortem PD brain sections. Neuroinflammation and the influenza A virus’s function in PD pathogenesis were convincingly demonstrated by the colocalization of influenza A and immune cells with caspase-cleaved Beclin-1 within the SNpc. It has been shown that the H5N1 influenza virus enhances α-synuclein phosphorylation and aggregation as it moves from the peripheral nervous system into the central nervous system (77).

Emerging RNA viruses that target the CNS cause cognitive consequences in survivors. Studies in people and animals infected with WNV, a re-emerging RNA virus linked to learning and memory disorders, demonstrated microglial-mediated synapse destruction in the hippocampus. Furthermore, CNS-resident memory T (TRM) cells activate microglia, which limits synapse regeneration and causes spatial learning deficits in WNV-recovered animals (78). Innate immune responses to emerging RNA viruses are becoming recognized as having substantial implications to neurologic sequelae, including memory impairments. Using a recovery model of WNV encephalitis it was found that, while macrophages deliver the antiviral and anti-neurogenic cytokine IL-1β during acute infection; viral recovery is associated with continued astrocyte inflammasome-mediated production of inflammatory levels of IL-1β, which is maintained by hippocampal astrogenesis via IL-1R1 signaling in neural stem cells (NSC). As a result, the absence of IL-1 signaling in NSC prevents abnormal astrogenesis, implying that only freshly produced astrocytes cause neurotoxicity by blocking synapse repair and enhancing spatial learning deficits (79, 80). In mice recovering from WNV or ZIKV infection, T cell-derived interferon-γ (IFN-γ) signaling in microglia causes spatial-learning defects through virus-target-specific mechanisms. Recovery from WNV infection resulted in presynaptic termini elimination with no repair, while recovery from ZIKV resulted in extensive neuronal apoptosis with loss of postsynaptic termini (81, 82).

### Viral hepatitis B and C neurological impairment

Systemic parenteral hepatitis is characterized by a wide range of neurological issues and symptoms caused by several immune illnesses (6). Pathological processes are caused by viral agents replicating within and outside of brain. Depending on the degree, neurological problems brought on by acute or chronic viral hepatitis may arise from the brain, spinal cord, or peripheral nervous system. From subclinical alterations to neurocritical situations, these symptoms can occur (83, 84). Viral particles’ direct neurotoxic effects on brain cells as well as the indirect effects of viruses’ influence on the immune system or from the use of antiviral medication are the causes of these disorders (85). Identifying the key neurological symptoms of individuals with viral hepatitis is critical for neurologists who treat these patients on a regular basis. This will make it easier to guarantee the quick implementation of diagnostic and treatment plans (83, 84).

Nevertheless, in the last few years, a growing body of research has investigated the relationship between the Hepatitis C virus and dementia (41, 86, 87). The mechanism underlying the emergence of dementia in viral hepatitis C patients is still unclear (41). Hepatitis viruses may be able to directly infect endothelial cells and get through the blood-brain barrier to reach the central nervous system The component molecules that viruses release during replication are known as pathogen-associated molecular patterns (PAMP). When the central nervous system is damaged in inflammatory infections, inflammatory mediators such TNF-α, IFN-γ, IL-1β, IL-6, IL-18, and chemokines are produced that promote neuronal death (88).

Parkinson’s disease pathogenesis in viral hepatitis is associated with the ability of hepatitis viruses to replicate in brain macrophages and microglial cells as well as their capability to pass the blood-brain barrier. Pro-inflammatory cytokines and chemokines are released more frequently as a result, which damages neurons and eventually results in their death (89, 90). Moreover, recent studies on rats have shown that the hepatitis virus depletes dopaminergic neurons in rodents’ brains (90, 91). Numerous studies have demonstrated that individuals with chronic viral hepatitis are more likely to develop PD (27, 29). Thus, a major population-based study conducted in Taiwan with 49,967 individuals who had viral hepatitis C revealed that this patient group is more prone to Parkinson’s disease than those who had no history of viral hepatitis (92). Previous studies have found similar results showing a considerable increased risk of Parkinson’s disease in individuals with viral hepatitis; nevertheless, to obtain more reliable data, the authors recommend doing further large-scale studies (28, 29, 93). Dementia, particularly Alzheimer’s disease, has been linked to HCV infection (94). According to a recent study, treating HCV infection with direct-acting antivirals (e.g., ledipasvir/sofosbuvir, elbasvir/grazoprevir, and glecaprevir/pibrentasvir) dramatically lowers the risk of death in individuals with dementia associated with AD (95). Furthermore, viruses play a significant role in the development of AD through promoting the accumulation of amyloid-β (Aβ) peptides in the brain (96). Previous research has shown that the blood-brain barrier permeability, which controls HCV infection and activity in the central nervous system, is influenced by the ApoE level, which is also strongly linked to the neuropsychiatric symptoms experienced by HCV-infected individuals (96, 97). Although evidence suggests that HCV infection is linked to CNS impairment, it is unclear if any HCV infection promotes AD etiology. Observational studies can be difficult to understand as the results may have been impacted by reverse causality and confounding factors.

### Human immunodeficiency virus type 1

In elderly HIV-1-positive patients receiving highly active antiretroviral therapy, age-related AD-like illness may be more likely to occur due to neurocognitive impairments associated with Aβ deposition and hyperphosphorylated Tau (98). HIV multiplies and contributes to neurodegeneration by affecting brain energetics at the cellular level, causing changes in overall brain metabolic homeostasis. Even though immunological dysfunction and dysregulation are typically attributed to the underlying pathophysiology of HIV infection, cognitive impairments associated with the virus have long been recognized. The spectrum of progressive neurological effects of infection includes asymptomatic neurocognitive impairments (ANI), moderate neurocognitive disorders (MND), and the more severe HIV-associated dementia (HAD) (99). According to estimates, 20–50% of HIV-positive individuals suffer from certain cognitive dysfunctions; these conditions are collectively known as HIV-associated neurological disorders (HAND). Functional status assessments and neuropsychological tests are used in the diagnosis of several disorders (100). HIV infection in the CNS is associated with activation of microglia and astrocytes, as well as the production of inflammatory and neurotoxic insults, all of which contribute to the neurodegeneration and cognitive impairment characteristic of HAND disease. Macrophages and microglia can release pro-inflammatory cytokines such as TNFα, IFNα, IL6, IL8, and IL1β, as well as chemokines such as CCL2, CCL5, and MIP-1β. These indications point to the presence of cellular reservoirs in the CNS established within 3 to 5 days of HIV-1 infection, which include three types of long-lived infected cells: astrocytes, monocyte lineage cells, and microglial cells (101). HIV enters the brain through infected CD4+ macrophages and lymphocytes, which permits the virus to transmigrate to the CNS’s perivascular spaces without being noticed by the immune system (102). The molecular and cellular mechanisms underpinning HIV-associated cognitive dysfunctions (HAND) are poorly understood, despite the prevalence of these disorders. These pathways are thought to combine the neurotoxic effects of HIV-associated proteins, indirect host factor involvement, and direct viral infection of CNS cells (103). Notably, it has been shown that the HIV viral proteins Tat and gp120 both increase viral entry into the central nervous system and modify the integrity of the blood-brain barrier. HIV transactivator of transcription, or Tat, is a viral regulatory protein that initiates viral transcription and is among the first HIV proteins to be generated upon infection (104).

Moreover, HIV-RNA in the cerebrospinal fluid (CSF) and viral replication in the CNS can occur in non-viremic people receiving combined antiretroviral therapy, a condition that can cause neurological harm like cognitive decline (105, 106). Despite a decrease in the occurrence of these disorders throughout the era of combined antiretroviral medication, the frequency of minor to severe HAND remains high, even in those who get sufficient treatment (100, 107). Neopterin levels in the CSF in HIV patients with viral suppression can actually be high (108). Neopterin is associated with both cognitive decline and phagocyte activity, suggesting a potential role for CNS phagocytes in neuronal damage and degeneration. CNS phagocytes express neurodegeneration associated molecules and are located topographically in inflammatory foci rich in reactive astrocytes. Neurodegenerative phagocytes appose neurons and consume synaptic material. Aberrant phagocyte activation may be responsible for the cognitive abnormalities seen in HAND. A notable histological characteristic of HAND is synaptic degeneration (109, 110). While persistent chronic inflammation is thought to contribute to cognitive decline, the molecular basis of CNS immune activation in the context of HAND remains little known. Because the population of HIV-positive people is aging, it is imperative to understand the processes behind these synaptic alterations in HIV in order to find new therapy targets to stop cognitive decline in HAND and other disorders (111).

### Influenza virus

Flu and neuropsychiatric disorders include encephalopathy, delirium, convulsions, and confusion are well-establishedly linked (112). Influenza infections during pregnancy have also been linked to a higher chance of schizophrenia or bipolar illness in the child (113). Numerous studies suggest that the neurological effects of influenza are caused by neuroinflammatory insult, which is primarily immune-mediated rather than the result of direct viral invasion of the CNS (114). Studies on animals have raised the possibility of a link between influenza and AD. In particular, these investigations have revealed increased microglial activity in the mouse hippocampal region, a place critical for the formation of new memories and an early stage in the pathophysiology of AD due to loss of neuronal cells (115). A follow-up study on mice was able to demonstrate a connection between influenza-induced hippocampus neuroinflammation and cognitive impairment (114).

There has been speculation of an infectious etiology, and some research has linked certain diseases to PD (116, 117). Whether influenza and Parkinson’s disease or parkinsonism are related has been debated for decades (118, 119). Influenza has been implicated in an outbreak of postencephalitic parkinsonism that happened from 1916 and 1930, right before and after the 1918 influenza pandemic (120, 121). The connection between influenza and Parkinson’s disease and parkinsonism has been extensively studied, and some of the results suggest that infections may be the root cause of some cases (91, 122). Neurotropic influenza-A virus-infected mice exhibit activation of microglia, inflammatory responses, and inclusions of α-Synuclein in dopaminergic neurons in an experimental setting (19). The primary protein component of Lewy bodies and Lewy neurites, α-syn, was in fact produced by dopaminergic cells expressing the H1N1 influenza virus, but not tau or Transactive response DNA binding protein 43 kDa (TDP-43) (123).

### SARS-CoV-2

Multiple sclerosis (MS), AD, and PD are neurodegenerative illnesses that are increasingly thought to be comorbidities in SARS-CoV-2-infected patients (124). Age dependence and co-morbidities like obesity, diabetes, and cardiovascular problems are among the many parallels between COVID-19 and PD. Furthermore, it is possible that COVID-19 will influence PD patient treatment practices and vice versa (125). Other common COVID-19 traits, such as fever, tension, and anxiety, may also negatively impact tremor, gait, and dyskinesias in PD, in addition to impairing the efficiency of L-Dopa (124). The functional relationship between AD and COVID-19 is becoming more and more evident. Like other neurodegenerative diseases, AD is considered a co-morbidity with COVID-19, meaning that having one condition usually makes the other worse (126). Neurodegeneration and neurocognitive impairment are associated with both situations with the buildup of amyloid precursor protein (APP) and activation of N-methyl-D-aspartate (NMDA)receptors. Furthermore, because these disorders share proinflammatory signaling cascades, neuronal cell death and dysfunction in both circumstances have been linked to microglial-mediated responses (127).

One of the largest RNA viruses is the SARS-CoV-2 virus. With the help of a complex array of accessory and nonstructural proteins, the virus is able to elude the innate immune system and replicate, translate, and exocytose as a fully functional virion. The single-stranded RNA that encodes 29 proteins includes the spike protein, which has the essential domains needed for binding to Angiotensin-converting enzyme 2 (ACE2). Furthermore, the possibility that these proteins have a role in the metabolic and molecular pathways of neurodegeneration is starting to gain more attention. Viruses or necessary protein components can be transported by extracellular vesicles to neurons in the substantia nigra, human cortical astrocytes, and microglia in addition to being directly absorbed by brain endothelium. This facilitates the faster formation of pathogenic fibrils (128). Liquid condensate can be produced by the intrinsically disordered SARS-CoV-2 nucleocapsid protein, which can even create harmful heteropolymers with RNA-binding proteins associated with neurodegenerative disease, such as TDP-43, fused-in sarcoma (FUS), and heterogeneous nuclear ribonucleoprotein A1 (hnRNP1A). More transmissible but less severe than the initial strain, the SARS-CoV-2 virus is continually evolving in response to the immune pressure imposed by very efficient vaccinations. Its potential long-term impacts on the brain system may therefore be a legacy of a global health crisis far more grave than acute disease (129). More severe SARS-CoV-2 and IAV infections are significantly correlated with aging-related proteostasis degradation in older people. A growing body of research indicates that the SARS-CoV-2 infection affects cognitive function over the long term and may eventually result in neurodegenerative diseases like AD (129–131). A number of pathways have been suggested, which are not mutually exclusive, while research to identify the exact mechanism(s) by which SARS-CoV-2 attacks the neurological system, both acutely and chronically, is underway (132, 133).

### Herpes simplex virus-1

Lifelong latent infections in sensory neurons are brought on by neurotropic herpesviruses. HSV-1 is a periodically reactivating virus that can enter the brain and cause encephalitis or create CNS latency. Many studies link AD and HSV-1. In fact, HSV-1 seropositivity appears to increase the risk of AD (134), and HSV-1 DNA can be detected in Aβ plaques (135). In animals and cellular models, reactivation of repeated HSV-1 infections results in the accumulation of hyperphosphorylated Tau and the AD biomarkers Aβ over time (136).

The ϵ4 genotype of APOE is a known risk factor for AD. In animal models, apoE ϵ4 appears to allow HSV1 latency in the brain much more and is more effective than apoE ϵ3 in promoting viral colonization of the brain following acute HSV1 infection (137). It was demonstrated that apoE ϵ4 was more common in the brains of AD patients who were HSV1-positive than HSV1-negative, and in those who had recurrent cold sores than in those who did not (138). These findings suggest that individuals with the apoE ϵ4 allele may be more susceptible to HSV’s effects on the brain.

### Human herpesvirus 6

HHV6 belongs to the β herpesvirus subfamily, which consists of two distinct species. It damages nerve cells and has been connected to a number of neurological disorders. The olfactory route allows HHV6 to enter the brain (139). In addition to AD, HHV6 is frequently seen in older, healthy brains. The HHV6 IgG antibodies reactivity of AD patients were significantly lower than that of normal controls. Although HHV6 might be linked to the genesis of AD, these findings might potentially point to a causal relationship or an opportunistic participant in neurodegeneration (140). A multiscale network analysis that includes late-onset AD-associated viromes and integrated genomic, transcriptomic, proteomic, and histological data from four distinct brain regions in human post-mortem tissue was used to demonstrate that AD patients had greater levels of HHV6A and human herpesvirus 7 than controls (141). There are regulatory relationships between viral abundance and APP metabolism modulators, including HHV-6A’s activation of APBB2, APPBP2, BIN1, BACE1, CLU, PICALM, and PSEN1. This suggests that specific virus species can cause neuropathology and Alzheimer’s disease (142).

### Other viruses involved in neurodegeneration

**Figure 2:**
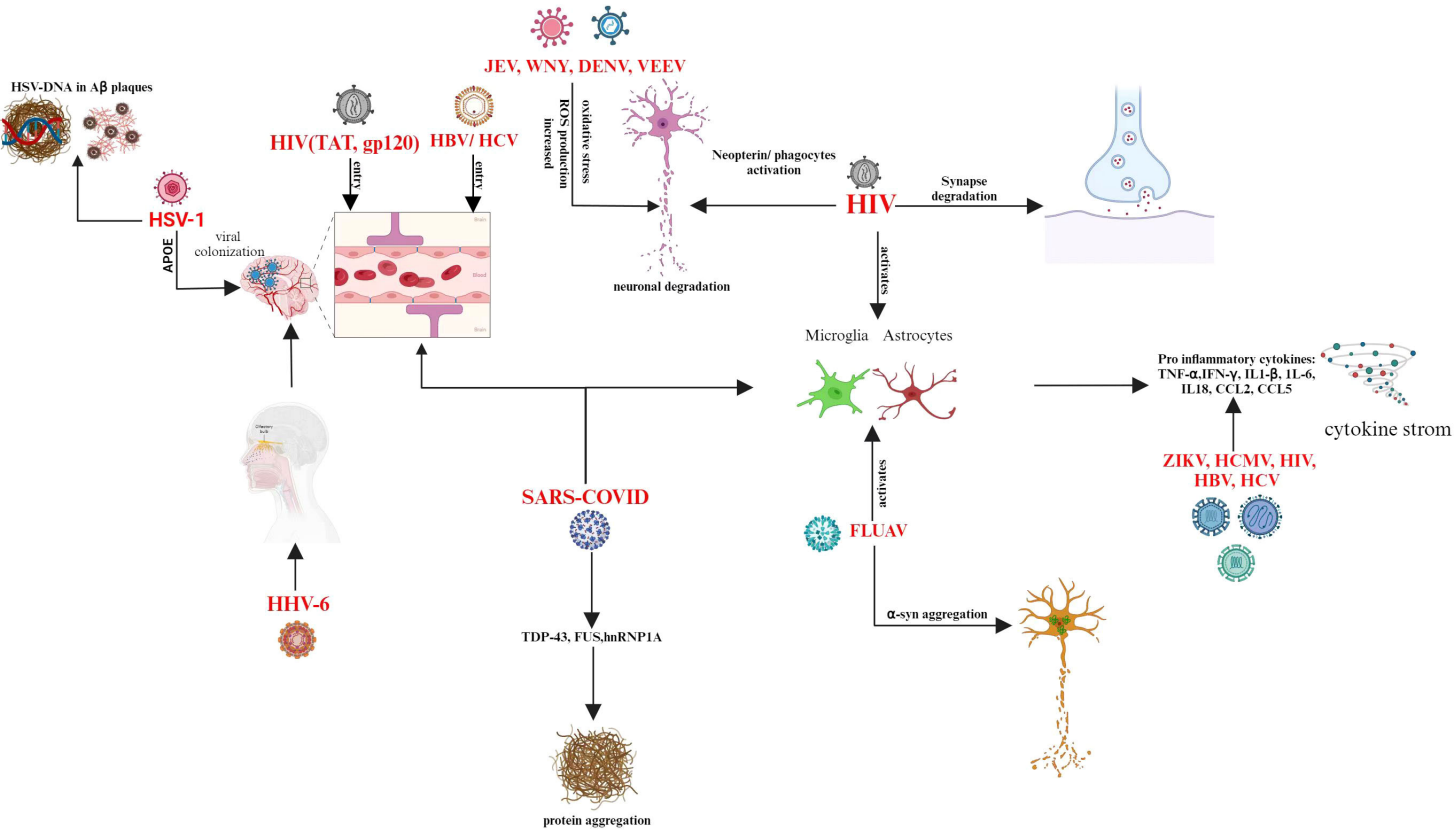
Entry routes of different viruses to infiltrate the CNS and induces neurodegeneration.

Recent research has unequivocally shown that a history of Epstein-Barr virus (EBV) infection is associated with a higher risk of developing multiple sclerosis (MS) (143). A motor neuron disease called ALS damages brain and spinal cord nerves. A build-up of RNA-binding proteins such as FUS or TDP-43, along with cytoplasmic mislocalization, are indicative of both frontotemporal dementia and ALS. An underlying viral infection that is ordinarily epigenetically repressed and incapable of replication is up-regulated in individuals with ALS (144). Enteroviruses in the brains and cerebrospinal fluid of individuals with ALS are a topic of discussion (48). However, mice infected with two enteroviruses developed an accumulation of TDP-43 and persistent inflammation (145). Mice infected with Theiler’s murine encephalitis virus (TMEV) developed an ALS-like phenotype with TDP-43 and FUS inclusions in their cytoplasm, which affected their motor neurons and glial cells (146). The Japanese encephalitis virus (JEV) can infect humans and cause Japanese encephalitis, which has a high death rate in severe cases and leaves 30 to 50 percent of survivors with severe, permanent neurological or mental repercussions (147). Increased production of reactive oxygen species (ROS) from JEV infection intensifies the death of neurons brought on by both mature and replication-incompetent viruses (148). Increased ROS production and decreased membrane fluidity in JEV-infected neuronal cells lead to serious cytopathic effects, which ultimately cause neuronal cell death (149). Neuronal cells infected with other members of the Flaviviridae family, such as West Nile virus (WNV) (70) and dengue virus type 2 (DENV-2) (150), also showed excessive O2.- production during viral infection, which resulted in host cell apoptosis. Different viruses adapt different routes to enter the CNS and causes neurodegeneration explained in **Figure 2**.

The Venezuelan equine encephalitis virus (VEEV) causes serious neurological abnormalities in 4–14% of patients, and fatal encephalitis in 1% of cases (151). Upon infection with VEEV, astrocytoma U87MG cells exhibit an abrupt rise in ROS levels (152). Deadly rabies virus (RABV) attacks the central nervous system (CNS), leading to encephalitis and ultimately mammalian death. Research has shown that RABV infection results in increased ROS production in mouse neuroblastoma cells (153). Inducing oxidative stress is a crucial role of the RABV viral component (154). Infection with the deadly RABV causes changes in cellular gene expression. RABV, like other neurodegenerative diseases, may be involved in neuronal death due to an imbalance in Ca2+ homeostasis. Due to the role of calcium homeostasis in dysregulation in neurodegenerative diseases and other pathophysiology, there is reason to assume that neurons that contain certain intracellular calcium-binding proteins have a greater capacity to buffer calcium, and therefore would be more resistant to degeneration (155). Oxidative stress is a major factor in the pathogenesis of neurodegeneration in viral infections of the central nervous system, as evidenced by elevated levels of free radicals and lipid peroxidation caused by neurotrophic viruses. **Table 2** lists the numerous population-based investigations that were carried out to determine the role of viruses in neurodegenerative diseases.

**Table 2:** Prospective cohort studies for involvement of viruses in NDs.

Virus	ND	Source	Year	References
HCV	PD	Taiwan National Health Insurance Research Database	2016	(92)
HCV/HBV	PD	Community-based integrated screening program in Taiwan	2015	(156)
Cytomegalovirus	PD	UK Biobank	2024	(157)
HBV/HCV	Dementia	Korean National Health Insurance Service	2021	(41)
HSV	AD/Dementia	Vasculature in Uppsala Seniors (PIVUS) cohort	2024	(37)
HSV/VZV	Dementia	Korean National Health Insurance Service	2017	(47)
HCV	Multiple sclerosis (MS)	Neurology department at Ain Shams University Hospital, Egypt	2023	(158)

## Possible mechanism of viral pathogenesis, inflammation and neurodegeneration

Neurotropic viruses are a type of newly and re-emerging infections that specifically target and damage the integrity of the CNS (159, 160). There are several distinctive ways in which they can enter the CNS, leading to a range of neurological symptoms (161). Viruses have a particular method in which they first enter the peripheral nervous system before migrating into the CNS via axon fibers (162). Neurotropic viruses employ a variety of techniques in addition to exploiting the peripheral nervous system to bypass host barrier defenses and directly infiltrate the central nervous system. For instance, immune cells like macrophages, monocytes, and dendritic cells can become infected by the Zika virus (ZIKV), human cytomegalovirus (HCMV), and human immunodeficiency virus (HIV), which then function as carriers to move the virus into the CNS (161, 163, 164). Moreover, it has been shown that viral infections stimulate the production of chemokines and pro-inflammatory chemicals like TNF-α, CCL2, CCL5, IL-6, and IL-8, which can trigger a cytokine storm. The systemic pro-inflammatory state impairs the blood-brain barrier, allowing more pro-inflammatory cytokines and viruses to enter the CNS. The cytokine storm at the nervous system level can cause neuronal death, activation of microglia, synaptic plasticity impairment, and neurotransmission dysfunction (161, 165). Viruses have the ability to activate astrocytes and microglia (166, 167), cause neuroinflammation (167), oxidative stress (168), immunological responses (159), protein aggregation (169), and upset the balance of microbes in the gut (170) after they have entered the central nervous system. Accumulating data has revealed a bidirectional relationship between the gut microbiome and CNS, known as the “microbiota-gut-brain axis.” Early microbiome changes were observed in preclinical Alzheimer’s disease (AD) and prodromal Parkinson’s disease (PD) patients (171, 172). These processes have the capacity to both initiate and exacerbate NDs.

Risk factors were recently analyzed with publicly available datasets from two large-scale population-based studies, UK Biobank and FinnGen. The UK Biobank contained twenty-two of the forty-five significant correlations between viral infections and NDs that were discovered in FinnGen. It’s interesting to see that the strongest hazard ratio was associated with viral encephalitis and AD (57). Additionally, utilizing virome analysis, nine viruses were shown to be present in various CNS brain tissues in patients with PD, with PD patients showing greater positive frequencies of viruses than patients in the control group (173). Remarkably, evidence from recent studies provide credence to the hypothesis that persons with viral illnesses may be less likely to develop NDs if they receive immunizations or antiviral drugs (123, 174). When considered collectively, these results provide credibility to the theory that viral infections raise the chances of NDs.

Viruses have evolved unique defense methods to evade host defense reactions. These mechanisms include autophagy disruption and additional interference with host antiviral signaling triggered by viral infection (175). In **Table 3** various mechanisms are summarized through which viruses cause neurodegeneration in AD and PD. Although some illnesses interfere with specific signaling pathways to prevent autophagosomes from fusing with lysosomes or lysosomal breakdown, autophagosomes can also serve as reproduction sites for viruses as they infect a host (202–204). Activation of autophagy by various viruses, including flaviviruses and enteroviruses, can promote virus spread by assembling and releasing infectious particles through autophagic vacuoles. In certain viral infection cases, such as poliovirus and coronavirus infection, autophagy induction by infected cells promotes the generation of double-membrane vesicles to enhance viral replication (205).

**Table 3:** Mechanisms adopted by viruses causing neurodegeneration in PD and AD.

Neurodegenerative disorder	Virus	Pathogenesis	Markers	References
PD	CVB3	cause neurons to develop α-syn-associated inclusion bodies, which may serve as a PD trigger	Elevated α-syn expression α-syn fibrils in damaged mitochondria	(16)
	HBV/HCV	Invade the central nervous system Dopaminergic neuron death	Elevated levels of TNF-a, IL-6, and IL-1b, IL-8, IL-29, IL-22	(176, 177)
	IAV	Increased mRNA levels of CD36, CD68, C1QA, and C3, together with a changed expression pattern of major histocompatibility complex classes I and II, CD80, and F4/80, indicating evolving synaptic pruning	Increased levels of IL-6 and IFN-γ, TNF	(114)
	West Nile virus (WNV)	Abnormalities in the basal ganglia, thalamus, and pons, mostly bilaterally, evident in T2 and DWI sequences	Damage to the substantia nigra Secretion of α-syn	(178–180)
	HIV	Dopaminergic basal ganglia damage Neuroinvasion	Tumor necrosis factor (TNF)-α, interleukin (IL)-6, and IL-1β production	(181, 182)
	JEV	Profound gliosis in the substantia nigra pars compacta (SNpc), similar to that seen in PD lower dopamine and norepinephrine levels in JEV-infected rats Dopaminergic and norepinephrinergic system impairment	Lower CSF concentrations of dopamine, norepinephrine and homovanillic acid	(30, 183, 184)
	IAV H5N1	Blocking protein degradation pathways Blocking of autophagosome formation and inhibition of autophagic flux	α-syn phosphorylation and aggregation	(19, 123)
AD	HHV-6A HHV-6B	Dysregulation of autophagy in neurons astrocytoma cells Neuroinflammation	Aβ deposition Increasing beta-amyloid and tau	(14, 134, 185–188)
	HIV,	Synaptic deficits Trojan horse mechanism	Aβ1–42 dysregulated Amyloid plaques in the CSF and blood	(189, 190)
	HCMV	Neuroinvasion	Aβ production Astrocyte reactivity	(191, 192)
	HHV-6/7	Neuroinflammation	Elevated tau, ApoE, and Aβ1–42 protein expression	(186, 193, 194)
	HSV-1	Accelerated Aβ deposition Gliosis Cognitive dysfunction	triggers the phosphorylation of Tau by activating protein kinase A (PKA) and glycogen synthase kinase 3β (GSK3β) Initiate the translation of β-site amyloid precursor protein cleaving enzyme 1 (BACE1) and the buildup of Aβ by activating RNA-activated protein kinase (PKR).	(195–197)
	Hepatitis viruses (HBV, HCV)	Infect endothelial cells directly and enter the central nervous system across the blood-brain barrier	Elevated levels of TNF-α, IFN-., IL-1ß, IL-6, IL-18, IL-10, IL-12 Elevated tau and amyloid beta-peptide levels	(41, 88)
	HHV-6A HHV-6B	Dysregulation of autophagy in neurons astrocytoma cells Neuroinflammation	Aβ deposition Increasing beta-amyloid and tau	(14, 185, 186)
PD/AD	SARS-CoV-2	Viral invasion Immune-mediated inflammation Endothelial dysfunction	Aggregation of Aß, α-syn, tau, and TDP-43	(198–201)

### Antiviral therapies in ND’s

Antivirals could be interesting alternative drug options for treating NDs. In cell culture, antivirals were able to decrease HSV-1-induced production of Aβ and phosphorylated Tau (206)Acyclovir, penciclovir, and foscarnet are anti-HSV1 antiviral medications that decreased Aβ and P-tau accumulation along with HSV1. The antiviral-induced decrease in Aβ is attributable to the reduced number of new viruses, and hence the reduction in viral spread. Since antiviral agents reduce greatly Aβ and P-tau accumulation in HSV1-infected cells, they would be suitable for treating AD with great advantage unlike current AD therapies, only the virus, not the host cell, would be targeted (206). Ribavirin is a low-molecular-weight nucleoside analogue and inhibitor of inosine monophosphate dehydrogenase that functions as a broad-spectrum antiviral drug against a variety of DNA and RNA viruses. Ribavirin is approved in the United States for the treatment of RSV infections and, when combined with interferon, for hepatitis C virus infections (207). However, studies have shown that, as compared to a placebo, oral ribavirin formulations do not improve virologic response or the treatment of chronic hepatitis C. As a result, ribavirin is not permitted for use as a monotherapy for hepatitis C (208). Moreover, despite divergent opinions in the literature, ribavirin has been shown to be efficient against HSV both on its own and in combination with acyclovir, where it has been shown to augment the effects of acyclovir (209). Activity of ribavirin against EV has been demonstrated *in vitro* (210). Hepatitis C, RSV, and HSV are among the infectious diseases that ribavirin effectively treats; AD has been connected to several of these infections (87, 209). In a clinical trial, the Apovir group’s CSF biomarker levels showed a decrease in Aβ42 over the duration of treatment (86).

The main antiviral drug used to treat HSV1 infections is called acyclovir (ACV); as expected, ACV dramatically reduces the number of HSV1 and the levels of Aβ and P-tau in HSV1-infected cells in culture (206). P-tau production is reliant on HSV1 replication and eventually drops to zero. Antibody formation is significantly decreased, but it depends, at least partially, on a previous phase of the cycle. Lower viral DNA replication is probably the cause of this decrease in viral dissemination. These results suggest that ACV might be helpful in the management of AD (211). Individuals who test positive for HSV have a higher likelihood of cognitive impairment, and antiviral drugs have a potent anti-HSV infection impact. Recent studies employing databases incorporating electronic health information have shown that HSV infections increase the risk of dementia, but antiviral medication treatment lowers this risk. In a trial including schizophrenia, the generic antiviral drug valacyclovir showed better memory improvement than a placebo (212). It has also been shown that acyclovir administration prevents HSV-1-induced neuronal death (213). When dexamethasone and acyclovir were given together, the impairments in spatial cognition were lessened. Together with microglia activation, this combination also decreased the levels of neuroinflammation markers as TNF-α and IL-6 (214). However, these effects happen only when acyclovir and dexamethasone are administered simultaneously.

Antiviral medication significantly reduces the risk of Parkinson’s disease in patients with viral hepatitis (25, 215). *In vitro* models showed that the anti-influenza drug oseltamivir phosphate inhibited the aggregation of α-synuclein caused by H1N1 (123). Antiviral medication has demonstrated promise in reducing the likelihood of HCV infection, which is a risk factor for PD. In patients, the incidence of PD with persistent HCV infection appeared to be lower when treated with interferon-based antiviral therapy (216). Anti-HIV drug maraviroc specifically inhibited CCR5, ameliorating tauopathies and Huntington’s disease (HD) in model mice (217).

Ever since the initial appearance of the acute respiratory coronavirus SARS-CoV-2, scientists have been searching for novel antiviral medications and repurposing those that have demonstrated efficacy against other coronaviruses. antiviral medication that could be applied in case of COVID-19 outbreak. PD, AD, and fatigue associated with multiple sclerosis have been shown to benefit from amantanes such as amantadine, rimantadine, and memantine. These conditions are all known comorbidities associated with COVID-19. Additionally, basic pharmacological studies conducted *in vitro* and *in vivo* have shown that amantadine can inhibit SARS-CoV-2 by down-regulating host-cell proteases, which impairs the release of the viral genome into the host cell, and by acting as an NMDA receptor antagonist, which prevents the acute lung injury and respiratory distress that are hallmarks of COVID-19 (124). Antiviral drugs like oseltamivir, which are frequently prescribed to treat influenza, have been demonstrated to significantly enhance parkinsonism and increase dyskinesia (218).

Antiviral drugs are now being tested for the treatment of ALS. Combination antiretroviral therapy lowers transcript levels of the HERV-K subtype HML-2, that has been demonstrated to be elevated in ALS (219). A Phase IIa clinical trial including ALS patients found that antiretroviral medication (effective against HERV-K HML-2) indicates a trend toward delayed disease progression in patients with virological response to the treatment (220). Even though the results were encouraging, more randomized controlled trials (RCTs) are now required to assess any potential advantages for NDs.

Additionally, the potential antiviral properties of bioflavonoids produced from Ginkgo biloba leaves, such as ginkgetin, isoginkgetin, and ginkgolic acid, were investigated. These substances have a well-established antiviral profile from earlier research (221). Ginkgetin has been shown to effectively block the synthesis of viral proteins and impede the replication of HSV-1, HSV-2, and the human cytomegalovirus (222). The important significance that traditional Chinese medicine plays in treating COVID-19 aftereffects has been acknowledged. Research has demonstrated that chalcones and flavonoids can prevent neurodegeneration, prolonged COVID-19 illness, and SARS-CoV-2 infection (223). The bioactive constituents of Ginkgo biloba extract, ginkgolides and bilobalide (BB), have demonstrated neuroprotective effects in AD via pathways including anti-excitotoxicity, anti-inflammatory, and anti-oxidative properties. Furthermore, by blocking the major protease of SARS-CoV-2, ginkgolides and BB may also have antiviral effects against COVID-19. But whether pure ginkgolides or BB are given over an extended period of time at potentially therapeutic doses is actually beneficial or harmful for treating COVID-19 and AD is still up for debate (223).

Different medications have demonstrated promise in alleviating the long-term clinical symptoms of COVID-19 and neurodegenerative disorders, despite the fact that there is presently no standardized treatment for COVID-19. One way to lessen the harmful impact on nerve cells is to either preserve internal Ca2+ homeostasis or prevent the long-term inflow of Ca2+ ions. By inhibiting the extrasynaptic N-methyl-D-aspartate receptors, N-methyl-D-aspartate antagonists such as amantadine and memantine can do this by reducing the long-term Ca2+ ion influx that contributes to neuronal excitotoxicity. Amantadine is an antiviral medication that has been demonstrated to help patients with PD with their altered motor behavior. It may also help with persistent fatigue. However, memantine might aid in the improvement of cognitive deficiencies. Overlooking these issues may result in neuronal death and the associated functional deficits (224). To ascertain the effectiveness and comprehend the molecular underpinnings of these drugs’ anti-coronavirus activity or inhibitory potential, more *in vitro* and *in vivo* research are required. Different antiviral drugs are in trials for neurodegenerative disorders explained in **Table 4**.

**Table 4:** Clinical trials of antiviral drugs against NDs.

ND	Virus	Anti-viral Drugs	Clinical trial	References
AD	Pleconaril (active on enteroviruses) ribavirin (active on several viruses)	Apovir	Phase IIa	(86)
AD	HSV	Valacyclovir	Phase II	https://clinicaltrials.gov/study/ NCT03282916
PD	Influenza	Amantadine	completed	https://clinicaltrials.gov/study/ NCT00632762
AD	HSV-1	Penciclovir		(206)
PD	HCV	Interferon-*α*		(216)
PD	HCV	Interferon-free direct-acting antiviral (DAA) therapy with ledipasvir (LDV) plus sofosbuvir (SOF)		(225)
ALS	HIV/AIDS	Combination Antiretroviral Therapy (Triumeq)	Phase IIa	https://clinicaltrials.gov/study/ NCT02868580
Schizophrenia	HSV-1	Valaciclovir (pro-drug of acyclovir)	Phase II	(226), https://clinicaltrials.gov/study/ NCT02008773
AD	HSV-1	Acyclovir		(214)
AD	HBV/HIV	Lamivudine/3TC	Phase I Phase II	https://clinicaltrials.gov/study/ NCT04552795
Mild Cognitive Impairment	HBV/HIV	Lamivudine/3TC	Phase II	https://clinicaltrials.gov/study/ NCT06519357
Amyotrophic Lateral Sclerosis (ALS)	HIV	Antiretroviral regimen approved to treat HIV	Phase I	https://clinicaltrials.gov/study/ NCT02437110
Multiple Seclerosis (MS)	Epstein-Barr virus (EBV)	Famciclovir	Phase II	https://clinicaltrials.gov/study/ NCT05283551
PD	HBV/HIV	Tenofovir Disoproxil Fumarate	Phase I	https://clinicaltrials.gov/study/ NCT06356662

### AAV gene therapy

In recent years, adeno-associated virus (AAV) has become the main vector for CNS gene therapy. AAV has already shown promising results in the clinic for a range of CNS ailments, including neuromuscular diseases, lysosomal storage disorders, and illnesses that are intractable with medicine. Gene therapy uses DNA or RNA as a pharmacological agent to produce gene products that permanently mute, repair, or modify endogenous genes. One “one-and-done” treatment method that can cross the blood-brain barrier is gene therapy (227) help prevent the long-term progression of neurological diseases (6). In recent years, gene therapies—like AAV-based therapy—have progressed from being the exclusive focus of preclinical research to being an effective form of treatment (228). AAV has the advantages of immunological privilege, high delivery efficiency, and specialized tissue or cell tropism in the CNS.

Regarding AAV-based gene therapy, the most clinically studied CNS condition is PD. PD is currently being studied using three different methods: glutamate decarboxylase (GAD)-inhibited glutamine synthesis as a neurotransmitter; aromatic amino acid decarboxylase, AADC-induced dopamine production; and glial cell line-derived neurotrophic factor (GDNF) in the substantia nigra to protect nigral neurons. However, the majority of AAV-based treatments are unable to treat pathologically complex diseases (229). To treat PD, AAV-based gene therapy vectors can increase dopamine levels in target cells (230). In PD primate model, intrastriatal infusion of an AAV vector containing the human aromatic l-amino acid decarboxylase (hAADC) gene results in robust gene expression (231). Alternatively, an AAV-based α-synuclein expression vector (AAV-PHP.B-GBA1) can be injected intravenously (IV) into the target neural parenchyma as an alternative to the more common injection of the mouse forebrain in PD gene therapy. Because of this, the vector was able to enter the brain parenchyma and propagate throughout it. This allowed the vector to target the central and peripheral nervous systems globally and restored animal behavior by reducing synucleinopathy (232).

More than one hundred clinical trials have involved Alzheimer’s patients. Other than immunotherapy, there is currently no medication that can impede the progression of Alzheimer’s disease in those with cognitive impairments. However, AAV-based gene therapy continues to be ineffective. The only experiment that was successfully completed used AAV2-driven nerve growth factor to reverse basal cholinergic neuronal dysfunction. Ten patients with mild-to-moderate AD were treated in a Phase I clinical trial with bilateral stereotactic injections of AAV2-nerve growth factor into the Meynert nucleus basalis without the use of immunosuppressive drugs (233). This medicine worked effectively, was safe, and was well tolerated. No side effects were reported. Another trial, a Phase II trial, used a higher dose, although the treatment and placebo groups’ outcomes in terms of brain metabolic or cognitive performance did not vary statistically (234). The autopsy results of the three cases showed that stereotactically injected AAV2 did not reach the nucleus basalis of Meynert due to restricted AAV2 diffusion; hence, no reliable conclusions could be drawn (235). Three other therapeutic modalities are the subject of clinical investigation, the results of which have not yet been made public: Intravenous or intrathecal telomerase (hTERT) delivery to lengthen telomeres; brain-derived neurotrophic factor administered via parenchymal delivery to minimize neuronal loss and promote synaptic reconstruction; and intra-CSF delivery of apolipoprotein E2 to restore protein expression in patients homozygous for apolipoprotein E4 (235).

Numerous novel issues highlight the need for further research, especially in the areas of safe delivery methods, well-understood immunological systems, cost-effective production procedures, targeted vectors, and further immune system suppression strategies. To extend AAV-based gene therapy from monogenic disorders to other diseases, we need to understand the whole phenotypic range of each disease and find objective biomarkers to capture the essential features of the condition. Ongoing research on the imaging of viral vectors is necessary to monitor the pharmacokinetics of viruses.

## Conclusion

CNS infection diagnosis and therapy are difficult but essential. There are either none or very few antiviral medications on the market now for treating viral infections of the central nervous system. A viral infection causes an imbalance between free radicals and antioxidants, which increases oxidative stress within cells and causes neuronal cells to undergo programmed death through apoptosis. In order to interfere with mitophagy and mitochondrial dynamics in their hosts, viruses work with the recycling machinery of the cell.

Viral disturbance of mitochondrial homeostasis alters neuronal metabolism and consequently affects brain function. When neurotropic viruses enter the brain, specific brain functions are harmed, neurotransmitter systems are changed, and pathological signs of NDs appear. An understanding of the neuropathogenesis of viral CNS infection may help in the creation of more efficient diagnosis and treatment plans by focusing on the molecular mechanisms underlying CNS infection. It might also be helpful in the search for new antiviral drugs, which are necessary to treat these neurotropic viral infections in an efficient manner.

## Glossary

AAV: Adeno-Associated Virus
ACE2: Angiotensin-Converting Enzyme 2
ACV: Acyclovir
AD: Alzheimer’s disease
ALS: Amyotrophic Lateral Sclerosis
ALS: Amyotrophic Lateral Sclerosis
ANI: Asymptomatic Neurocognitive Impairments
ApoE: Apolipoprotein E
APOE ϵ4: Apolipoprotein E ε4 allele
APP: Amyloid Precursor Protein
Aβ: Amyloid-β
BACE1: β-Site Amyloid Precursor Protein Cleaving Enzyme 1
CNS: Central Nervous System
CSF: Cerebrospinal Fluid
CVB3: Coxsackievirus B3
DAA: Direct-Acting Antiviral
DENV-2: Dengue Virus Type 2
EBV: Epstein-Barr virus
EBV: Epstein-Barr Virus
EVs: Enteroviruses
GAD: Glutamate Decarboxylase
GDNF: Glial Cell Line-Derived Neurotrophic Factor
GSK3β: Glycogen Synthase Kinase 3β
GWAS: Genome-Wide Association Studies
HAD: HIV-Associated Dementia
HAND: HIV-Associated Neurological Disorders
HBV: Hepatitis B virus
HCMV: Human Cytomegalovirus
HCV: Hepatitis C virus
HHV: Human Herpesvirus
HHV6: Human Herpesvirus 6
HIV: Human Immunodeficiency Virus
HSV: Herpes simplex virus
HSV-1: Herpes Simplex Virus-1
IAV: Influenza A virus
IV: Intravenously
JEV: Japanese encephalitis virus
JEV: Japanese Encephalitis Virus’
LDV: Ledipasvir
MND: Moderate Neurocognitive Disorders
MS: Multiple sclerosis
MSA: Multiple Systems Atrophy
NDs: Neurodegenerative diseases
NMDA: N-methyl-D-aspartate
PD: Parkinson’s disease
PKA: Protein Kinase A
PKR: RNA-Activated Protein Kinase
RABV: Rabies Virus
ROS: Reactive Oxygen Species
SNpc: Substantia Nigra Pars Compacta
SOF: Sofosbuvir
TMEV: Theiler’s Murine Encephalitis Virus
VEEV: Venezuelan Equine Encephalitis Virus
VZV: Varicella-Zoster Virus
WEV: Western equine virus
WNV: West Nile Virus
WNV: West Nile virus
ZIKV: Zika virus
α-syn: α-synuclein.

## References

[1] Zhou L Miranda-Saksena M Saksena NK. Viruses and neurodegeneration. Virol J. (2013) 10:172. doi: 10.1186/1743-422X-10-172 23724961 PMC3679988

[2] Wang YA Kammenga JE Harvey SC. Genetic variation in neurodegenerative diseases and its accessibility in the model organism Caenorhabditis elegans. Hum Genomics. (2017) 11:1–10. doi: 10.1186/S40246-017-0108-4 28545550 PMC5445269

[3] Mirza Z Kamal M Buzenadah A Al-Qahtani M Karim S. Establishing genomic/transcriptomic links between Alzheimer’s disease and type 2 diabetes mellitus by meta-analysis approach. CNS Neurol Disord Drug Targets. (2014) 13:501–16. doi: 10.2174/18715273113126660154 24059308

[4] Griffin WST. Inflammation and neurodegenerative diseases. Am J Clin Nutr. (2006) 83(2):470S–4S. doi: 10.1093/AJCN/83.2.470S 16470015

[5] Muravchick S Levy RJ. Clinical implications of mitochondrial dysfunction. Anesthesiology. (2006) 105:819–37. doi: 10.1097/00000542-200610000-00029 17006082

[6] Nicolson GL. Chronic bacterial and viral infections in neurodegenerative and neurobehavioral diseases. Lab Med. (2008) 39:291. doi: 10.1309/96M3BWYP42L11BFU

[7] Calabrese G Molzahn C Mayor T. Protein interaction networks in neurodegenerative diseases: From physiological function to aggregation. J Biol Chem. (2022) 298(7):102062. doi: 10.1016/J.JBC.2022.102062 35623389 PMC9234719

[8] Sengupta U Kayed R. Amyloid β, Tau, and α-Synuclein aggregates in the pathogenesis, prognosis, and therapeutics for neurodegenerative diseases. Prog Neurobiol. (2022) 214:102270. doi: 10.1016/J.PNEUROBIO.2022.102270 35447272

[9] Wang Q Zheng J Pettersson S Reynolds R Tan EK. The link between neuroinflammation and the neurovascular unit in synucleinopathies. Sci Adv. (2023) 9(7):eabq1141. doi: 10.1126/SCIADV.ABQ1141 36791205 PMC9931221

[10] Scheltens P De Strooper B Kivipelto M Holstege H Chételat G Teunissen CE. Alzheimer’s disease. Lancet. (2021) 397:1577–90. doi: 10.1016/S0140-6736(20)32205-4 PMC8354300 33667416

[11] Liu H Zheng Q Yuan J Gao Y Wang T Zhang H. Modulating SQSTM1/p62-dependent selective autophagy of neurons by activating Nrf2 with multifunctional nanoparticles to eliminate α-synuclein aggregates and boost therapy of Parkinson’s disease. Nano Today. (2023) 49:101770. doi: 10.1016/J.NANTOD.2023.101770

[12] Vaquer-Alicea J Diamond MI. Propagation of protein aggregation in neurodegenerative diseases. Annu Rev Biochem. (2019) 88:785–810. doi: 10.1146/ANNUREV-BIOCHEM-061516-045049 30917002

[13] Balchin D Hayer-Hartl M Hartl FU. *In vivo* aspects of protein folding and quality control. Science. (2016) 353(6294):aac4354. doi: 10.1126/SCIENCE.AAC4354 27365453

[14] Aviner R Frydman J. Proteostasis in viral infection: unfolding the complex virus-chaperone interplay. Cold Spring Harb Perspect Biol. (2020) 12(3):a034090. doi: 10.1101/CSHPERSPECT.A034090 30858229 PMC7050591

[15] Shelkovnikova TA An H Skelt L Tregoning JS Humphreys IR Buchman VL. Antiviral immune response as a trigger of FUS proteinopathy in amyotrophic lateral sclerosis. Cell Rep. (2019) 29:4496–4508.e4. doi: 10.1016/J.CELREP.2019.11.094 31875556 PMC6941233

[16] Park SJ Jin U Park SM. Interaction between coxsackievirus B3 infection and α-synuclein in models of Parkinson’s disease. PloS Pathog. (2021) 17(10):e1010018. doi: 10.1371/JOURNAL.PPAT.1010018 34695168 PMC8568191

[17] Jin U Park SJ Lee BG Kim JB Kim SJ Joe EH. Critical roles of parkin and PINK1 in coxsackievirus B3-induced viral myocarditis. Microbes Infect. (2023) 25:105211. doi: 10.1016/J.MICINF.2023.105211 37574181

[18] Chemparthy DT Kannan M Gordon L Buch S Sil S. Alzheimer’s-like pathology at the crossroads of HIV-associated neurological disorders. Vaccines (Basel). (2021) 9(8):930. doi: 10.3390/VACCINES9080930 34452054 PMC8402792

[19] Jang H Boltz D Sturm-Ramirez K Shepherd KR Jiao Y Webster R. Highly pathogenic H5N1 influenza virus can enter the central nervous system and induce neuroinflammation and neurodegeneration. Proc Natl Acad Sci U S A. (2009) 106:14063–8. doi: 10.1073/PNAS.0900096106 PMC2729020 19667183

[20] Cocoros NM Svensson E Szépligeti SK Vestergaard SV Szentkúti P Thomsen RW. Long-term risk of parkinson disease following influenza and other infections. JAMA Neurol. (2021) 78:1461–70. doi: 10.1001/JAMANEUROL.2021.3895 PMC8546623 34694344

[21] Maramattom BV Philips G. Acute parkinsonism with west nile virus infection. Ann Indian Acad Neurol. (2023) 26:801. doi: 10.4103/AIAN.AIAN_539_23 38022432 PMC10666871

[22] Lenka A Kamat A Mittal SO. Spectrum of movement disorders in patients with neuroinvasive west nile virus infection. Mov Disord Clin Pract. (2019) 6:426. doi: 10.1002/MDC3.12806 31392241 PMC6660229

[23] Bantle CM Phillips AT Smeyne RJ Rocha SM Olson KE Tjalkens RB. Infection with mosquito-borne alphavirus induces selective loss of dopaminergic neurons, neuroinflammation and widespread protein aggregation. NPJ Parkinson’s Dis. (2019) 5:1–15. doi: 10.1038/s41531-019-0090-8 31531390 PMC6744428

[24] Schultz DR Barthal JS Garrett C. Western equine encephalitis with rapid onset of parkinsonism. Neurology. (1977) 27:1095–6. doi: 10.1212/WNL.27.11.1095 563006

[25] Selim R Gordon SC Zhou Y Zhang T Lu M Daida YG. Impact of hepatitis C treatment status on risk of Parkinson’s disease and secondary parkinsonism in the era of direct-acting antivirals. J Viral Hepat. (2023) 30:544–50. doi: 10.1111/JVH.13826 36872452

[26] Golabi P Otgonsuren M Sayiner M Arsalla A Gogoll T Younossi ZM. The prevalence of parkinson disease among patients with hepatitis C infection. Ann Hepatol. (2017) 16:342–8. doi: 10.5604/01.3001.0009.8588 28425403

[27] Wijarnpreecha K Chesdachai S Jaruvongvanich V Ungprasert P. Hepatitis C virus infection and risk of Parkinson’s disease: a systematic review and meta-analysis. Eur J Gastroenterol Hepatol. (2018) 30:9–13. doi: 10.1097/MEG.0000000000000991 29049127

[28] Lilach G Fogel-Grinvald H Israel S. Hepatitis B and C virus infection as a risk factor for Parkinson’s disease in Israel-A nationwide cohort study. J Neurol Sci. (2019) 398:138–41. doi: 10.1016/J.JNS.2019.01.012 30710864

[29] Choi HY Mai TH Kim KA Cho H Ki M. Association between viral hepatitis infection and Parkinson’s disease: A population-based prospective study. J Viral Hepat. (2020) 27:1171–8. doi: 10.1111/JVH.13346 32558154

[30] Ogata A Tashiro K Nukuzuma S Nagashima K Hall WW. A rat model of Parkinson’s disease induced by Japanese encephalitis virus. J Neurovirol. (1997) 3:141–7. doi: 10.3109/13550289709015803 9111176

[31] Tadokoro K Ohta Y Sato K Maeki T Sasaki R Takahashi Y. A Japanese encephalitis patient presenting with parkinsonism with corresponding laterality of magnetic resonance and dopamine transporter imaging findings. Internal Med. (2018) 57:2243. doi: 10.2169/INTERNALMEDICINE.0337-17 29526949 PMC6120820

[32] Camacho-Soto A Faust I Racette BA Clifford DB Checkoway H Nielsen SS. Herpesvirus infections and risk of parkinson’s disease. Neurodegener Dis. (2020) 20:97–103. doi: 10.1159/000512874 33461199 PMC8552529

[33] Tunnicliffe L Weil RS Breuer J Rodriguez-Barradas MC Smeeth L Rentsch CT. Herpes zoster and risk of incident parkinson’s disease in US veterans: A matched cohort study. Movement Disord. (2024) 39:438–44. doi: 10.1002/MDS.29701 PMC10922272 38226430

[34] Hsieh JC Lue KH Lee YL. Parkinson-like syndrome as the major presenting symptom of Epstein–Barr virus encephalitis. Arch Dis Child. (2002) 87:358–8. doi: 10.1136/ADC.87.4.358 PMC1763051 12244024

[35] Tiwari D Mittal N Jha HC. Unraveling the links between neurodegeneration and Epstein-Barr virus-mediated cell cycle dysregulation. Curr Res Neurobiol. (2022) 3:100046. doi: 10.1016/J.CRNEUR.2022.100046 36685766 PMC9846474

[36] Itzhaki RF. Overwhelming evidence for a major role for herpes simplex virus type 1 (HSV1) in alzheimer’s disease (AD); underwhelming evidence against. Vaccines (Basel). (2021) 9(6):679. doi: 10.3390/VACCINES9060679 34205498 PMC8234998

[37] Vestin E Boström G Olsson J Elgh F Lind L Kilander L. Herpes simplex viral infection doubles the risk of dementia in a contemporary cohort of older adults: A prospective study. J Alzheimers Dis. (2024) 97:1841–50. doi: 10.3233/JAD-230718 PMC10894565 38306033

[38] Calcagno A Celani L Trunfio M Orofino G Imperiale D Atzori C. Alzheimer dementia in people living with HIV. Neurol Clin Pract. (2021) 11:e627. doi: 10.1212/CPJ.0000000000001060 34840876 PMC8610525

[39] Hussain H Fadel A Garcia E Michel G Saadoon ZF Fernandes A. HIV and dementia. Microbe. (2024) 2:100052. doi: 10.1016/J.MICROB.2024.100052

[40] Romanescu C Schreiner TG Mukovozov I. The role of human herpesvirus 6 infection in alzheimer’s disease pathogenicity—A theoretical mosaic. J Clin Med. (2022) 11:3061. doi: 10.3390/JCM11113061 35683449 PMC9181317

[41] Choi HG Soh JS Lim JS Sim SY Lee SW. Association between dementia and hepatitis B and C virus infection. Medicine. (2021) 100:E26476. doi: 10.1097/MD.0000000000026476 34398003 PMC8294892

[42] Tan CH Chang MC Tsai WF Chuang WL Huang JF Lin ZY. Different profiles of neurocognitive impairment in patients with hepatitis B and C virus infections. Sci Rep. (2022) 12:1–11. doi: 10.1038/s41598-022-14736-3 35739162 PMC9226189

[43] Huang L Wang Y Tang Y He Y Han Z. Lack of causal relationships between chronic hepatitis C virus infection and alzheimer’s disease. Front Genet. (2022) 13:828827/PDF. doi: 10.3389/FGENE.2022.828827/PDF 35356425 PMC8959984

[44] Chiu WC Chen PC. PIN79 hepatitis C virus infection increases the risk of alzheimer’S diseases. Value Health. (2012) 15:A399. doi: 10.1016/j.jval.2012.08.1146

[45] Sim KY An J Bae SE Yang T Ko GH Hwang JR. Alzheimer’s disease risk associated with changes in Epstein-Barr virus nuclear antigen 1-specific epitope targeting antibody levels. J Infect Public Health. (2024) 17:102462. doi: 10.1016/J.JIPH.2024.05.050 38824738

[46] Cairns DM Itzhaki RF Kaplan DL. Potential involvement of varicella zoster virus in alzheimer’s disease via reactivation of quiescent herpes simplex virus type 1. J Alzheimers Dis. (2022) 88:1189–200. doi: 10.3233/JAD-220287 35754275

[47] Shin E Chi SA Chung TY Kim HJ Kim K Lim DH. The associations of herpes simplex virus and varicella zoster virus infection with dementia: a nationwide retrospective cohort study. Alzheimer’s Res Ther. (2024) 16:1–10. doi: 10.1186/S13195-024-01418-7/TABLES/2 38475873 PMC10935826

[48] Xue YC Feuer R Cashman N Luo H. Enteroviral infection: The forgotten link to amyotrophic lateral sclerosis? Front Mol Neurosci. (2018) 11:63/PDF. doi: 10.3389/FNMOL.2018.00063/PDF 29593492 PMC5857577

[49] Cabrera JR Rodríguez-Izquierdo I Jiménez JL Muñoz-Fernández MÁ. Analysis of ALS-related proteins during herpes simplex virus-2 latent infection. J Neuroinflammation. (2020) 17:1–15. doi: 10.1186/S12974-020-02044-4/FIGURES/6 33287823 PMC7722435

[50] Bjornevik K Münz C Cohen JI Ascherio A. Epstein–Barr virus as a leading cause of multiple sclerosis: mechanisms and implications. Nat Rev Neurol. (2023) 19:160–71. doi: 10.1038/s41582-023-00775-5 36759741

[51] Khalesi Z Tamrchi V Razizadeh MH Letafati A Moradi P Habibi A. Association between human herpesviruses and multiple sclerosis: A systematic review and meta-analysis. Microb Pathog. (2023) 177:106031. doi: 10.1016/J.MICPATH.2023.106031 36775211

[52] Elhalag RH Motawea KR Talat NE Rouzan SS Reyad SM Elsayed SM. Herpes Zoster virus infection and the risk of developing dementia: A systematic review and meta-analysis. Medicine. (2023) 102:E34503. doi: 10.1097/MD.0000000000034503 37904465 PMC10615483

[53] Lotz SK Blackhurst BM Reagin KL Funk KE. Microbial infections are a risk factor for neurodegenerative diseases. Front Cell Neurosci. (2021) 15:691136/PDF. doi: 10.3389/FNCEL.2021.691136/PDF 34305533 PMC8292681

[54] Griciuc A Tanzi RE. The role of innate immune genes in Alzheimer’s disease. Curr Opin Neurol. (2021) 34:228–36. doi: 10.1097/WCO.0000000000000911 PMC7954128 33560670

[55] Parhizkar S Holtzman DM. “APOE mediated neuroinflammation and neurodegeneration in Alzheimer’s disease.” In Seminars in immunology. Vol. 59. Academic Press (2022). doi: 10.1016/J.SMIM.2022.101594 PMC9411266 35232622

[56] Li C Liu J Lin J Shang H. COVID-19 and risk of neurodegenerative disorders: A Mendelian randomization study. Transl Psychiatry. (2022) 12(1):283. doi: 10.1038/S41398-022-02052-3 35835752 PMC9281279

[57] Levine KS Leonard HL Blauwendraat C Iwaki H Johnson N Bandres-Ciga S. Virus exposure and neurodegenerative disease risk across national biobanks. Neuron. (2023) 111:1086–1093.e2. doi: 10.1016/J.NEURON.2022.12.029 36669485 PMC10079561

[58] Shouman S Hesham N Salem TZ. Viruses and neurodegeneration: a growing concern. J Trans Med. (2025) 23:1–21. doi: 10.1186/S12967-024-06025-6 PMC11727702 39800721

[59] Mathew S Faheem M Ibrahim SM Iqbal W Rauff B Fatima K. Hepatitis C virus and neurological damage. World J Hepatol. (2016) 8:545. doi: 10.4254/WJH.V8.I12.545 27134702 PMC4840160

[60] Leblanc P Vorberg IM. Viruses in neurodegenerative diseases: More than just suspects in crimes. PloS Pathog. (2022) 18:e1010670. doi: 10.1371/JOURNAL.PPAT.1010670 35925897 PMC9352104

[61] Wang WY Tan MS Yu JT Tan L. Role of pro-inflammatory cytokines released from microglia in Alzheimer’s disease. Ann Transl Med. (2015) 3(10):136. doi: 10.3978/J.ISSN.2305-5839.2015.03.49 26207229 PMC4486922

[62] Wongchitrat P Chanmee T Govitrapong P. Molecular mechanisms associated with neurodegeneration of neurotropic viral infection. Mol Neurobiol. (2023) 61:2881–903. doi: 10.1007/S12035-023-03761-6 PMC11043213 37946006

[63] Zhao YJ Xu KF Shu FX Zhang F. Neurotropic virus infection and neurodegenerative diseases: Potential roles of autophagy pathway. CNS Neurosci Ther. (2023) 30:e14548. doi: 10.1111/CNS.14548 38082503 PMC11163195

[64] Bramlett HM Dietrich WD. Long-term consequences of traumatic brain injury: current status of potential mechanisms of injury and neurological outcomes. J Neurotrauma. (2015) 32:1834. doi: 10.1089/NEU.2014.3352 25158206 PMC4677116

[65] Onisiforou A Spyrou GM. Identification of viral-mediated pathogenic mechanisms in neurodegenerative diseases using network-based approaches. Brief Bioinform. (2021) 22:bbab141. doi: 10.1093/BIB/BBAB141 34237135 PMC8574625

[66] Kavouras J Prandovszky E Valyi-Nagy K Kovacs SK Tiwari V Kovacs M. Herpes simplex virus type 1 infection induces oxidative stress and the release of bioactive lipid peroxidation by-products in mouse P19N neural cell cultures. J Neurovirol. (2007) 13:416–25. doi: 10.1080/13550280701460573 17994426

[67] Thangaraj A Periyasamy P Liao K Bendi VS Callen S Pendyala G. HIV-1 TAT-mediated microglial activation: role of mitochondrial dysfunction and defective mitophagy. Autophagy. (2018) 14:1596. doi: 10.1080/15548627.2018.1476810 29966509 PMC6135576

[68] Cho YE Lee MH Song BJ. Neuronal cell death and degeneration through increased nitroxidative stress and tau phosphorylation in HIV-1 transgenic rats. PloS One. (2017) 12:e0169945. doi: 10.1371/JOURNAL.PONE.0169945 28107387 PMC5249108

[69] Srivastava R Kalita J Khan MY Misra UK. Free radical generation by neurons in rat model of Japanese encephalitis. Neurochem Res. (2009) 34:2141–6. doi: 10.1007/S11064-009-0008-7 19495969

[70] Verma S Molina Y Lo YY Cropp CB Arai S Nakano CM. Role of oxidative stress in west nile virus (WNV)- induced apoptosis. FASEB J. (2006) 20:A1073–A1073. doi: 10.1096/FASEBJ.20.5.A1073-B

[71] Fonseka CL Hardman CS Woo J Singh R Nahler J Yang J. Dengue virus co-opts innate type 2 pathways to escape early control of viral replication. Commun Biol. (2022) 5:735. doi: 10.1038/S42003-022-03682-5 35869167 PMC9306424

[72] Jan J-T Chen B-H Ma S-H Liu C-I Tsai H-P Wu H-C. Potential dengue virus-triggered apoptotic pathway in human neuroblastoma cells: arachidonic acid, superoxide anion, and NF-kappaB are sequentially involved. J Virol. (2000) 74:8680–91. doi: 10.1128/JVI.74.18.8680-8691.2000 PMC116379 10954569

[73] Zhou Y Hou Y Shen J Mehra R Kallianpur A Culver DA. A network medicine approach to investigation and population-based validation of disease manifestations and drug repurposing for COVID-19. PloS Biol. (2020) 18:e3000970. doi: 10.1371/JOURNAL.PBIO.3000970 33156843 PMC7728249

[74] Lippi A Domingues R Setz C Outeiro TF Krisko A. SARS-coV-2: at the crossroad between aging and neurodegeneration. Movement Disord. (2020) 35:716–20. doi: 10.1002/MDS.28084 PMC7262312 32291797

[75] Vanderheiden A Hill JD Jiang X Deppen B Bamunuarachchi G Soudani N. Vaccination reduces central nervous system IL-1β and memory deficits after COVID-19 in mice. Nat Immunol. (2024) 25:1158–71. doi: 10.1038/S41590-024-01868-Z PMC13148132 38902519

[76] Soung AL Vanderheiden A Nordvig AS Sissoko CA Canoll P Mariani MB. COVID-19 induces CNS cytokine expression and loss of hippocampal neurogenesis. Brain. (2022) 145:4193–201. doi: 10.1093/BRAIN/AWAC270 PMC9452175 36004663

[77] Rohn TT Catlin LW. Immunolocalization of influenza A virus and markers of inflammation in the human Parkinson’s disease brain. PloS One. (2011) 6(5):e20495. doi: 10.1371/JOURNAL.PONE.0020495 21655265 PMC3105060

[78] Rosen SF Soung AL Yang W Ai S Kanmogne M Davé VA. Single-cell RNA transcriptome analysis of CNS immune cells reveals CXCL16/CXCR6 as maintenance factors for tissue-resident T cells that drive synapse elimination. Genome Med. (2022) 14:1–20. doi: 10.1186/S13073-022-01111-0/FIGURES/7 36153630 PMC9509564

[79] Soung AL Dave VA Garber C Tycksen ED Vollmer LL Klein RS. Corrigendum to: “IL-1 reprogramming of adult neural stem cells limits neurocognitive recovery after viral encephalitis by maintaining a proinflammatory state. Brain Behav Immun. (2022) 102:387. doi: 10.1016/j.bbi.2021.12.024 34695572 PMC10236567

[80] Garber C Vasek MJ Vollmer LL Sun T Jiang X Klein RS. Astrocytes decrease adult neurogenesis during virus-induced memory dysfunction via IL-1. Nat Immunol. (2018) 19:151–61. doi: 10.1038/S41590-017-0021-Y PMC5786497 29292385

[81] Garber C Soung A Vollmer LL Kanmogne M Last A Brown J. T cells promote microglia-mediated synaptic elimination and cognitive dysfunction during recovery from neuropathogenic flaviviruses. Nat Neurosci. (2019) 22:1276–88. doi: 10.1038/S41593-019-0427-Y PMC6822175 31235930

[82] Vasek MJ Garber C Dorsey D Durrant DM Bollman B Soung A. A complement-microglial axis drives synapse loss during virus-induced memory impairment. Nature. (2016) 534:538–43. doi: 10.1038/NATURE18283 PMC5452615 27337340

[83] Schwendimann RN Minagar A. Liver disease and neurology. Continuum (Minneap Minn). (2017) 23:762–77. doi: 10.1212/CON.0000000000000486 28570328

[84] Ferro JM Viana P Santos P. Management of neurologic manifestations in patients with liver disease. Curr Treat Options Neurol. (2016) 18:1–17. doi: 10.1007/S11940-016-0419-0 27314429

[85] Pawełczyk A. Consequences of extrahepatic manifestations of hepatitis C viral infection (HCV). Postepy Hig Med Dosw (Online). (2016) 70:349–59. doi: 10.5604/17322693.1199988 27117111

[86] Lindblom N Lindquist L Westman J Aström M Bullock R Hendrix S. Potential virus involvement in alzheimer’s disease: results from a phase IIa trial evaluating apovir, an antiviral drug combination. J Alzheimers Dis Rep. (2021) 5:413. doi: 10.3233/ADR-210301 34189413 PMC8203284

[87] Chiu WC Tsan YT Tsai SL Chang CJ Wang JD Chen PC. Hepatitis C viral infection and the risk of dementia. Eur J Neurol. (2014) 21(8):1068–e59. doi: 10.1111/ENE.12317 24313931

[88] Sochocka M Zwolińska K Leszek J. The infectious etiology of alzheimer’s disease. Curr Neuropharmacol. (2017) 15:996. doi: 10.2174/1570159X15666170313122937 28294067 PMC5652018

[89] Abushouk AI El-Husseny MWA Magdy M Ismail A Attia A Ahmed H. Evidence for association between hepatitis C virus and Parkinson’s disease. Neurol Sci. (2017) 38:1913–20. doi: 10.1007/S10072-017-3077-4 28780707

[90] Benito-León J. Viral hepatitis and the risk of Parkinson disease. Neurology. (2017) 88:1596–7. doi: 10.1212/WNL.0000000000003853 28356459

[91] Smeyne RJ Noyce AJ Byrne M Savica R Marras C. Infection and risk of parkinson’s disease. J Parkinsons Dis. (2021) 11:31–43. doi: 10.3233/JPD-202279 33361610 PMC7990414

[92] Tsai HH Liou HH Muo CH Lee CZ Yen RF Kao CH. Hepatitis C virus infection as a risk factor for Parkinson disease: A nationwide cohort study. Neurology. (2016) 86:840–6. doi: 10.1212/WNL.0000000000002307 26701382

[93] Abushouk AI Negida A Ahmed H Abdel-Daim MM. Neuroprotective mechanisms of plant extracts against MPTP induced neurotoxicity: Future applications in Parkinson’s disease. BioMed Pharmacother. (2017) 85:635–45. doi: 10.1016/J.BIOPHA.2016.11.074 27890431

[94] Lin HC Xirasagar S Lee HC Huang CC Chen CH. Association of Alzhemier’s disease with hepatitis C among patients with bipolar disorder. PloS One. (2017) 12(6):e0179312. doi: 10.1371/JOURNAL.PONE.0179312 28622343 PMC5473552

[95] Tran L Jung J Carlin C Lee S Zhao C Feldman R. Use of direct-acting antiviral agents and survival among medicare beneficiaries with dementia and chronic hepatitis C. J Alzheimers Dis. (2021) 79:71. doi: 10.3233/JAD-200949 33216031 PMC7855832

[96] Yamazaki Y Zhao N Caulfield TR Liu CC Bu G. Apolipoprotein E and Alzheimer disease: pathobiology and targeting strategies. Nat Rev Neurol. (2019) 15:501–18. doi: 10.1038/S41582-019-0228-7 PMC7055192 31367008

[97] Sheridan DA Bridge SH Crossey MME Felmlee DJ Thomas HC Neely RDG. Depressive symptoms in chronic hepatitis C are associated with plasma apolipoprotein E deficiency. Metab Brain Dis. (2014) 29:625–34. doi: 10.1007/S11011-014-9520-9 24615429

[98] Fulop T Witkowski JM Larbi A Khalil A Herbein G Frost EH. Does HIV infection contribute to increased beta-amyloid synthesis and plaque formation leading to neurodegeneration and Alzheimer’s disease? J Neurovirol. (2019) 25:634–47. doi: 10.1007/S13365-019-00732-3 30868421

[99] Kanki PJ Hopper JR Essex M. The origins of HIV-1 and HTLV-4/HIV-2. Ann N Y Acad Sci. (1987) 511:370–5. doi: 10.1111/J.1749-6632.1987.TB36265.X 2894192

[100] Antinori A Arendt G Becker JT Brew BJ Byrd DA Cherner M. Updated research nosology for HIV-associated neurocognitive disorders. Neurology. (2007) 69:1789–99. doi: 10.1212/01.WNL.0000287431.88658.8B PMC4472366 17914061

[101] López AB Penedo MA Rivera-Baltanas T Pérez-Rodríguez D Alonso-Crespo D Fernández-Pereira C. Microglia: the real foe in HIV-1-associated neurocognitive disorders? Biomedicines. (2021) 9:925. doi: 10.3390/BIOMEDICINES9080925 34440127 PMC8389599

[102] Gras G Kaul M. Molecular mechanisms of neuroinvasion by monocytes-macrophages in HIV-1 infection. Retrovirology. (2010) 7:1–11. doi: 10.1186/1742-4690-7-30/FIGURES/1 20374632 PMC2864195

[103] Smith LK Kuhn TB Chen J Bamburg JR. HIV associated neurodegenerative disorders: A new perspective on the role of lipid rafts in gp120-mediated neurotoxicity. Curr HIV Res. (2018) 16:258. doi: 10.2174/1570162X16666181003144740 30280668 PMC6398609

[104] Das AT Harwig A Berkhout B. The HIV-1 tat protein has a versatile role in activating viral transcription. J Virol. (2011) 85:9506. doi: 10.1128/JVI.00650-11 21752913 PMC3165771

[105] Fois AF Brew BJ. The potential of the CNS as a reservoir for HIV-1 infection: implications for HIV eradication. Curr HIV/AIDS Rep. (2015) 12:299–303. doi: 10.1007/S11904-015-0257-9 25869939

[106] Canestri A Lescure FX Jaureguiberry S Moulignier A Amiel C Marcelin AG. Discordance between cerebral spinal fluid and plasma HIV replication in patients with neurological symptoms who are receiving suppressive antiretroviral therapy. Clin Infect Dis. (2010) 50:773–8. doi: 10.1086/650538 20100092

[107] Simioni S Cavassini M Annoni JM Rimbault Abraham A Bourquin I Schiffer V. Cognitive dysfunction in HIV patients despite long-standing suppression of viremia. AIDS. (2010) 24:1243–50. doi: 10.1097/QAD.0B013E3283354A7B 19996937

[108] Motta I Allice T Romito A Ferrara M Ecclesia S Imperiale D. Cerebrospinal fluid viral load and neopterin in HIV-positive patients with undetectable viraemia. Antivir Ther. (2017) 22:539–43. doi: 10.3851/IMP3140 28198350

[109] Levine AJ Soontornniyomkij V Achim CL Masliah E Gelman BB Sinsheimer JS. Multilevel analysis of neuropathogenesis of neurocognitive impairment in HIV. J Neurovirol. (2016) 22:431. doi: 10.1007/S13365-015-0410-7 26637429 PMC4893344

[110] Sá MJ Madeira MD Ruela C Volk B Mota-Miranda A Paula-Barbosa MM. Dendritic changes in the hippocampal formation of AIDS patients: a quantitative Golgi study. Acta Neuropathol. (2004) 107:97–110. doi: 10.1007/S00401-003-0781-3 14605830

[111] Borrajo A Spuch C Penedo MA Olivares JM Agís-Balboa RC. Important role of microglia in HIV-1 associated neurocognitive disorders and the molecular pathways implicated in its pathogenesis. Ann Med. (2021) 53:43. doi: 10.1080/07853890.2020.1814962 32841065 PMC7877929

[112] Popescu CP Florescu SA Lupulescu E Zaharia M Tardei G Lazar M. Neurologic complications of influenza B virus infection in adults, Romania. Emerg Infect Dis. (2017) 23:574. doi: 10.3201/EID2304.161317 28322689 PMC5367398

[113] Brown AS Begg MD Gravenstein S Schaefer CA Wyatt RJ Bresnahan M. Serologic evidence of prenatal influenza in the etiology of schizophrenia. Arch Gen Psychiatry. (2004) 61:774–80. doi: 10.1001/ARCHPSYC.61.8.774 15289276

[114] Jurgens HA Amancherla K Johnson RW. Influenza infection induces neuroinflammation, alters hippocampal neuron morphology, and impairs cognition in adult mice. J Neurosci. (2012) 32:3958–68. doi: 10.1523/JNEUROSCI.6389-11.2012 PMC3353809 22442063

[115] Sadasivan S Zanin M O’Brien K Schultz-Cherry S Smeyne RJ. Induction of microglia activation after infection with the non-neurotropic A/CA/04/2009 H1N1 influenza virus. PloS One. (2015) 10(4):e0124047. doi: 10.1371/JOURNAL.PONE.0124047 25861024 PMC4393251

[116] Limphaibool N Iwanowski P Holstad MJV Kobylarek D Kozubski W. Infectious etiologies of parkinsonism: pathomechanisms and clinical implications. Front Neurol. (2019) 10:652. doi: 10.3389/FNEUR.2019.00652 31275235 PMC6593078

[117] Olsen LK Dowd E McKernan DP. A role for viral infections in Parkinson’s etiology? Neuronal Signal. (2018) 2(2):NS20170166. doi: 10.1042/NS20170166 32714585 PMC7373231

[118] Henry J Smeyne RJ Jang H Miller B Okun MS. Parkinsonism and neurological manifestations of influenza throughout the 20th and 21st centuries. Parkinsonism Relat Disord. (2010) 16:566–71. doi: 10.1016/J.PARKRELDIS.2010.06.012 PMC4684089 20650672

[119] Moore G. Influenza and parkinson’s disease. Public Health Rep. (1977) 92:79–80. 834846 PMC1431968

[120] Poskanzer DC Schwab RS. COHORT ANALYSIS OF PARKINSON’S SYNDROME: EVIDENCE FOR A SINGLE ETIOLOGY RELATED TO SUBCLINICAL INFECTION ABOUT 1920. J Chronic Dis. (1963) 16:961–73. doi: 10.1016/0021-9681(63)90098-5 14066517

[121] Dourmashkin RR. What caused the 1918–30 epidemic of encephalitis lethargica? J R Soc Med. (1997) 90:515. doi: 10.1177/014107689709000916 9370993 PMC1296535

[122] Estupinan D Nathoo S Okun MS. The demise of poskanzer and schwab’s influenza theory on the pathogenesis of parkinson’s disease. Parkinsons Dis. (2013) 2013:167843. doi: 10.1155/2013/167843 23853734 PMC3693163

[123] Marreiros R Müller-Schiffmann A Trossbach SV Prikulis I Hänsch S Weidtkamp-Peters S. Disruption of cellular proteostasis by H1N1 influenza A virus causes α-synuclein aggregation. Proc Natl Acad Sci U S A. (2020) 117:6741–51. doi: 10.1073/PNAS.1906466117 PMC7104400 32152117

[124] Butterworth RF. Adamantanes for the treatment of neurodegenerative diseases in the presence of SARS-CoV-2. Front Neurosci. (2023) 17:1128157/BIBTEX. doi: 10.3389/FNINS.2023.1128157/BIBTEX 36968489 PMC10031118

[125] Prasad S Holla VV Neeraja K Surisetti BK Kamble N Yadav R. Parkinson’s disease and COVID-19: perceptions and implications in patients and caregivers. Movement Disord. (2020) 35:912. doi: 10.1002/MDS.28088 32304118 PMC7264599

[126] Xia X Wang Y Zheng J. COVID-19 and Alzheimer’s disease: how one crisis worsens the other. Transl Neurodegener. (2021) 10(1):15. doi: 10.1186/S40035-021-00237-2 33941272 PMC8090526

[127] Butterworth RF. Memantine for the treatment of alzheimer’s disease: novel mechanisms and future opportunities. Neurol Neurorehabilitation. (2022) 5:17–20. doi: 10.37532/22.4.2.17-20

[128] Justo Arevalo S Castillo-Chávez A Uribe Calampa CS Zapata Sifuentes D Huallpa CJ Landa Bianchi G. What do we know about the function of SARS-CoV-2 proteins? Front Immunol. (2023) 14:1249607. doi: 10.3389/FIMMU.2023.1249607 37790934 PMC10544941

[129] Strong MJ. SARS-CoV-2, aging, and Post-COVID-19 neurodegeneration. J Neurochem. (2023) 165:115–30. doi: 10.1111/JNC.15736 PMC9877664 36458986

[130] Liu N Jiang X Li H. The viral hypothesis in Alzheimer’s disease: SARS-CoV-2 on the cusp. Front Aging Neurosci. (2023) 15:1129640/PDF. doi: 10.3389/FNAGI.2023.1129640/PDF 37009449 PMC10050697

[131] Krey L Huber MK Höglinger GU Wegner F. Can SARS-coV-2 infection lead to neurodegeneration and parkinson’s disease? Brain Sci. (2021) 11:1654. doi: 10.3390/BRAINSCI11121654 34942956 PMC8699589

[132] Crook H Raza S Nowell J Young M Edison P. Long covid—mechanisms, risk factors, and management. BMJ. (2021) 374. doi: 10.1136/BMJ.N1648 34312178

[133] Jha NK Ojha S Jha SK Dureja H Singh SK Shukla SD. Evidence of coronavirus (CoV) pathogenesis and emerging pathogen SARS-coV-2 in the nervous system: A review on neurological impairments and manifestations. J Mol Neurosci. (2021) 71:2192–209. doi: 10.1007/S12031-020-01767-6 PMC7814864 33464535

[134] Wozniak MA Shipley SJ Combrinck M Wilcock GK Itzhaki RF. Productive herpes simplex virus in brain of elderly normal subjects and Alzheimer’s disease patients. J Med Virol. (2005) 75:300–6. doi: 10.1002/JMV.20271 15602731

[135] Wozniak M Mee AP Itzhaki RF. Herpes simplex virus type 1 DNA is located within Alzheimer’s disease amyloid plaques. J Pathol. (2009) 217:131–8. doi: 10.1002/PATH.2449 18973185

[136] Marcocci ME Napoletani G Protto V Kolesova O Piacentini R Li Puma DD. Herpes simplex virus-1 in the brain: the dark side of a sneaky infection. Trends Microbiol. (2020) 28:808–20. doi: 10.1016/J.TIM.2020.03.003 32386801

[137] Burgos JS Ramirez C Sastre I Valdivieso F. Effect of apolipoprotein E on the cerebral load of latent herpes simplex virus type 1 DNA. J Virol. (2006) 80:5383–7. doi: 10.1128/JVI.00006-06 PMC1472141 16699018

[138] Itzhaki RF Lin WR Shang D Wilcock GK Faragher B Jamieson GA. Herpes simplex virus type 1 in brain and risk of Alzheimer’s disease. Lancet. (1997) 349:241–4. doi: 10.1016/S0140-6736(96)10149-5 9014911

[139] Harberts E Yao K Wohler JE Maric D Ohayon J Henkin R. Human herpesvirus-6 entry into the central nervous system through the olfactory pathway. Proc Natl Acad Sci U S A. (2011) 108:13734. doi: 10.1073/PNAS.1105143108 21825120 PMC3158203

[140] Westman G Blomberg J Yun Z Lannfelt L Ingelsson M Eriksson BM. Decreased HHV-6 igG in alzheimer’s disease. Front Neurol. (2017) 8:40/BIBTEX. doi: 10.3389/FNEUR.2017.00040/BIBTEX 28265256 PMC5316842

[141] Readhead B Haure-Mirande JV Funk CC Richards MA Shannon P Haroutunian V. Multiscale analysis of three independent Alzheimer’s cohorts reveals disruption of molecular, genetic, and clinical networks by Human herpesvirus. Neuron. (2018) 99:64. doi: 10.1016/J.NEURON.2018.05.023 29937276 PMC6551233

[142] Readhead B Haure-Mirande JV Funk CC Richards MA Shannon P Haroutunian V. Multiscale analysis of independent alzheimer’s cohorts finds disruption of molecular, genetic, and clinical networks by human herpesvirus. Neuron. (2018) 99:64–82.e7. doi: 10.1016/J.NEURON.2018.05.023 29937276 PMC6551233

[143] Bjornevik K Cortese M Healy BC Kuhle J Mina MJ Leng Y. Longitudinal analysis reveals high prevalence of Epstein-Barr virus associated with multiple sclerosis. Science. (2022) 375:296–301. doi: 10.1126/SCIENCE.ABJ8222 35025605

[144] Douville R Liu J Rothstein J Nath A. Identification of active loci of a human endogenous retrovirus in neurons of patients with amyotrophic lateral sclerosis. Ann Neurol. (2011) 69:141–51. doi: 10.1002/ANA.22149 PMC3052883 21280084

[145] Fung G Shi J Deng H Hou J Wang C Hong A. Cytoplasmic translocation, aggregation, and cleavage of TDP-43 by enteroviral proteases modulate viral pathogenesis. Cell Death Differentiation. (2015) 22:2087–97. doi: 10.1038/cdd.2015.58 PMC4816113 25976304

[146] Masaki K Sonobe Y Ghadge G Pytel P Roos RP. TDP-43 proteinopathy in Theiler’s murine encephalomyelitis virus infection. PloS Pathog. (2019) 15(2):e1007574. doi: 10.1371/JOURNAL.PPAT.1007574 30742696 PMC6390522

[147] Unni SK Růžek D Chhatbar C Mishra R Johri MK Singh SK. Japanese encephalitis virus: from genome to infectome. Microbes Infect. (2011) 13:312–21. doi: 10.1016/J.MICINF.2011.01.002 21238600

[148] Lin RJ Liao CL Lin YL. Replication-incompetent virions of Japanese encephalitis virus trigger neuronal cell death by oxidative stress in a culture system. J Gen Virol. (2004) 85:521–33. doi: 10.1099/VIR.0.19496-0 14769909

[149] Mishra MK Ghosh D Duseja R Basu A. Antioxidant potential of Minocycline in Japanese Encephalitis Virus infection in murine neuroblastoma cells: correlation with membrane fluidity and cell death. Neurochem Int. (2009) 54:464–70. doi: 10.1016/J.NEUINT.2009.01.022 19428790

[150] Jan J-T Chen B-H Ma S-H Liu C-I Tsai H-P Wu H-C. Potential dengue virus-triggered apoptotic pathway in human neuroblastoma cells: arachidonic acid, superoxide anion, and NF-κB are sequentially involved. J Virol. (2000) 74:8680–91. doi: 10.1128/JVI.74.18.8680-8691.2000/ASSET/EE619D90-E640-447C-8F71-BBD3E36FA736/ASSETS/GRAPHIC/JV1800201009.JPEG PMC116379 10954569

[151] Ronca SE Dineley KT Paessler S. Neurological sequelae resulting from encephalitic alphavirus infection. Front Microbiol. (2016) 7:959/PDF. doi: 10.3389/FMICB.2016.00959/PDF 27379085 PMC4913092

[152] Keck F Brooks-Faulconer T Lark T Ravishankar P Bailey C Salvador-Morales C. Altered mitochondrial dynamics as a consequence of Venezuelan Equine encephalitis virus infection. Virulence. (2017) 8:1849–66. doi: 10.1080/21505594.2016.1276690 PMC5810500 28075229

[153] Kammouni W Wood H Saleh A Appolinario CM Fernyhough P Jackson AC. Rabies virus phosphoprotein interacts with mitochondrial Complex I and induces mitochondrial dysfunction and oxidative stress. J Neurovirol. (2015) 21:370–82. doi: 10.1007/S13365-015-0320-8 25698500

[154] Kammouni W Wood H Jackson AC. Serine residues at positions 162 and 166 of the rabies virus phosphoprotein are critical for the induction of oxidative stress in rabies virus infection. J Neurovirol. (2017) 23:358–68. doi: 10.1007/S13365-016-0506-8 27995576

[155] Kanu B Kia GSN Aimola IA Korie GC Tekki IS. Rabies virus infection is associated with alterations in the expression of parvalbumin and secretagogin in mice brain. Metab Brain Dis. (2021) 36:1267. doi: 10.1007/S11011-021-00717-4 33783673 PMC8008021

[156] Wu WYY Kang KH Chen SLS Chiu SYH Yen AMF Fann JCY. Hepatitis C virus infection: a risk factor for Parkinson’s disease. J Viral Hepat. (2015) 22:784–91. doi: 10.1111/JVH.12392 25608223

[157] Ma X Liao Z Tan H Wang K Feng C Xing P. The association between cytomegalovirus infection and neurodegenerative diseases: a prospective cohort using UK Biobank data. EClinicalMedicine. (2024) 74. doi: 10.1016/j.eclinm.2024.102757 PMC11327475 39157287

[158] Khater SS Elnaser AA Abdallah D Zamzam D Elaziz DA. Is hepatitis C virus incriminated in pathogenesis of multiple sclerosis? Mult Scler Relat Disord. (2023) 80:105220. doi: 10.1016/J.MSARD.2023.105220

[159] Vazquez C Jurado KA. Neurotropic RNA virus modulation of immune responses within the central nervous system. Int J Mol Sci. (2022) 23(7):4018. doi: 10.3390/IJMS23074018 35409387 PMC8999457

[160] McMillan RE Wang E Carlin AF Coufal NG. Human microglial models to study host-virus interactions. Exp Neurol. (2023) 363:114375. doi: 10.1016/J.EXPNEUROL.2023.114375 36907350 PMC10521930

[161] Tan LY Komarasamy TV James W Balasubramaniam VRMT. Host molecules regulating neural invasion of zika virus and drug repurposing strategy. Front Microbiol. (2022) 13:743147/PDF. doi: 10.3389/FMICB.2022.743147/PDF 35308394 PMC8931420

[162] McGavern DB Kang SS. Illuminating viral infections in the nervous system. Nat Rev Immunol. (2011) 11:318–29. doi: 10.1038/NRI2971 PMC5001841 21508982

[163] Ene L. Human immunodeficiency virus in the brain-culprit or facilitator? Infect Dis. (2018) 11:117863371775268. doi: 10.1177/1178633717752687 PMC5815409 29467577

[164] Haspot F Lavault A Sinzger C Sampaio KL Stierhof YD Pilet P. Human cytomegalovirus entry into dendritic cells occurs via a macropinocytosis-like pathway in a pH-independent and cholesterol-dependent manner. PloS One. (2012) 7(4):e34795. doi: 10.1371/JOURNAL.PONE.0034795 22496863 PMC3322158

[165] Yuan S Jiang SC Zhang ZW Fu YF Hu J Li ZL. Quantification of cytokine storms during virus infections. Front Immunol. (2021) 12:659419/PDF. doi: 10.3389/FIMMU.2021.659419/PDF 34079547 PMC8165266

[166] Quincozes-Santos A Bobermin LD Costa NLF Thomaz NK Almeida RR de S Beys-da-Silva WO. The role of glial cells in Zika virus-induced neurodegeneration. Glia. (2023) 71:1791–803. doi: 10.1002/GLIA.24353 36866453

[167] Patrycy M Chodkowski M Krzyzowska M. Role of microglia in herpesvirus-related neuroinflammation and neurodegeneration. Pathogens. (2022) 11(7):809. doi: 10.3390/PATHOGENS11070809 35890053 PMC9324537

[168] Duarte LF Farías MA Álvarez DM Bueno SM Riedel CA González PA. Herpes simplex virus type 1 infection of the central nervous system: Insights into proposed interrelationships with neurodegenerative disorders. Front Cell Neurosci. (2019) 13:46/PDF. doi: 10.3389/FNCEL.2019.00046/PDF 30863282 PMC6399123

[169] Ge T Yuan Y. Herpes simplex virus infection increases beta-amyloid production and induces the development of alzheimer’s disease. BioMed Res Int. (2022) 2022(1):8804925. doi: 10.1155/2022/8804925 36093396 PMC9453006

[170] Follmer C. Viral infection-induced gut dysbiosis, neuroinflammation, and α-synuclein aggregation: updates and perspectives on COVID-19 and neurodegenerative disorders. ACS Chem Neurosci. (2020) 11:4012–6. doi: 10.1021/ACSCHEMNEURO.0C00671 33244974

[171] Loh JS Mak WQ Tan LKS Ng CX Chan HH Yeow SH. Microbiota–gut–brain axis and its therapeutic applications in neurodegenerative diseases. Signal Transduction Targeted Ther. (2024) 9:1–53. doi: 10.1038/s41392-024-01743-1 PMC10869798 38360862

[172] Ashique S Mohanto S Ahmed MG Mishra N Garg A Chellappan DK. Gut-brain axis: A cutting-edge approach to target neurological disorders and potential synbiotic application. Heliyon. (2024) 10:e34092. doi: 10.1016/J.HELIYON.2024.E34092 39071627 PMC11279763

[173] Deng L Fu P Ding L Duan X Feng S Peng Y. Virome analysis provides new insights into the association between viruses and Parkinson’s disease. J Med Virol. (2023) 95(1):e28111. doi: 10.1002/JMV.28111 36042689

[174] Bukhbinder AS Ling Y Hasan O Jiang X Kim Y Phelps KN. Risk of alzheimer’s disease following influenza vaccination: A claims-based cohort study using propensity score matching. J Alzheimers Dis. (2022) 88:1061–74. doi: 10.3233/JAD-220361 PMC9484126 35723106

[175] Zhou A Zhang W Dong X Liu M Chen H Tang B. The battle for autophagy between host and influenza A virus. Virulence. (2022) 13:46–59. doi: 10.1080/21505594.2021.2014680 34967267 PMC9794007

[176] Yaow CYL Hong ASY Chong NZY Chong RIH Mai AS Tan EK. Risk of Parkinson’s disease in hepatitis B and C populations: a systematic review and meta-analysis. J Neural Transm. (2024) 131:609–16. doi: 10.1007/S00702-023-02705-7/FIGURES/7 37899363

[177] Zhang Y Cobleigh MA Lian JQ Huang CX Booth CJ Bai XF. A proinflammatory role for interleukin-22 in the immune response to hepatitis B virus. Gastroenterology. (2011) 141:1897–906. doi: 10.1053/J.GASTRO.2011.06.051 PMC3199295 21708106

[178] Sejvar JJ. Clinical manifestations and outcomes of west nile virus infection. Viruses. (2014) 6:606. doi: 10.3390/V6020606 24509812 PMC3939474

[179] Schafernak KT Bigio EH. West Nile virus encephalomyelitis with polio-like paralysis & nigral degeneration. Can J Neurol Sci. (2006) 33:407–10. doi: 10.1017/S0317167100005370 17168167

[180] Beatman EL Massey A Shives KD Burrack KS Chamanian M Morrison TE. Alpha-synuclein expression restricts RNA viral infections in the brain. J Virol. (2015) 90:2767–82. doi: 10.1128/JVI.02949-15 PMC4810656 26719256

[181] Clifford DB. Human immunodeficiency virus–associated dementia. Arch Neurol. (2000) 57:321–4. doi: 10.1001/ARCHNEUR.57.3.321 10714656

[182] Dehner LF Spitz M Pereira JS. Parkinsonism in HIV infected patients during antiretroviral therapy - data from a Brazilian tertiary hospital. Braz J Infect Dis. (2016) 20:499–501. doi: 10.1016/J.BJID.2016.05.008 27449286 PMC9425509

[183] Hamaue N Ogata A Terado M Ohno K Kikuchi S Sasaki H. Brain catecholamine alterations and pathological features with aging in Parkinson disease model rat induced by Japanese encephalitis virus. Neurochem Res. (2006) 31:1451–5. doi: 10.1007/S11064-006-9197-5 17103330

[184] Leta V Urso D Batzu L Lau YH Mathew D Boura I. Viruses, parkinsonism and Parkinson’s disease: the past, present and future. J Neural Transm. (2022) 129:1119. doi: 10.1007/S00702-022-02536-Y 36036863 PMC9422946

[185] Eimer WA Vijaya Kumar DK Navalpur Shanmugam NK Rodriguez AS Mitchell T Washicosky KJ. Alzheimer’s disease-associated β-amyloid is rapidly seeded by herpesviridae to protect against brain infection. Neuron. (2018) 99:56–63.e3. doi: 10.1016/J.NEURON.2018.06.030 30001512 PMC6075814

[186] Bortolotti D Gentili V Rotola A Caselli E Rizzo R. HHV-6A infection induces amyloid-beta expression and activation of microglial cells. Alzheimers Res Ther. (2019) 11:1–11. doi: 10.1186/S13195-019-0552-6/FIGURES/5 31831060 PMC6909659

[187] Harris SA Harris EA. Molecular mechanisms for herpes simplex virus type 1 pathogenesis in Alzheimer’s disease. Front Aging Neurosci. (2018) 10:48/BIBTEX. doi: 10.3389/FNAGI.2018.00048/BIBTEX 29559905 PMC5845560

[188] De Chiara G Marcocci ME Civitelli L Argnani R Piacentini R Ripoli C. APP processing induced by herpes simplex virus type 1 (HSV-1) yields several APP fragments in human and rat neuronal cells. PloS One. (2010) 5:13989. doi: 10.1371/JOURNAL.PONE.0013989 PMC2981559 21085580

[189] Canet G Dias C Gabelle A Simonin Y Gosselet F Marchi N. HIV neuroinfection and Alzheimer’s disease: Similarities and potential links? Front Cell Neurosci. (2018) 12:307/BIBTEX. doi: 10.3389/FNCEL.2018.00307/BIBTEX 30254568 PMC6141679

[190] Clifford DB Fagan AM Holtzman DM Morris JC Teshome M Shah AR. CSF biomarkers of Alzheimer disease in HIV-associated neurologic disease. Neurology. (2009) 73:1982. doi: 10.1212/WNL.0B013E3181C5B445 19907013 PMC2790234

[191] Blanck G Huda TI Chobrutskiy BI Chobrutskiy A. CMV as a factor in the development of Alzheimer’s disease? Med Hypotheses. (2023) 178:111140. doi: 10.1016/J.MEHY.2023.111140

[192] Barbian HJ Lurain NS Bennett DA Al-Harthi L Hannah Barbian CJ. HCMV infection induces AD pathology in astrocytes. vitro. Alzheimer’s Dementia. (2020) 16:e039591. doi: 10.1002/ALZ.039591

[193] De Francesco MA. Herpesviridae, neurodegenerative disorders and autoimmune diseases: what is the relationship between them? Viruses. (2024) 16(1):133. doi: 10.3390/V16010133 38257833 PMC10818483

[194] Rizzo R Bortolotti D Gentili V Rotola A Bolzani S Caselli E. KIR2DS2/KIR2DL2/HLA-C1 haplotype is associated with alzheimer’s disease: implication for the role of herpesvirus infections. J Alzheimers Dis. (2019) 67:1379–89. doi: 10.3233/JAD-180777 30689576

[195] Wozniak MA Frost AL Itzhaki RF. Alzheimer’s disease-specific tau phosphorylation is induced by herpes simplex virus type 1. J Alzheimers Dis. (2009) 16:341–50. doi: 10.3233/JAD-2009-0963 19221424

[196] ILL-Raga G Palomer E Wozniak MA Ramos-Fernández E Bosch-Morató M Tajes M. Activation of PKR causes amyloid ß-peptide accumulation via de-repression of BACE1 expression. PloS One. (2011) 6(6):e21456. doi: 10.1371/JOURNAL.PONE.0021456 21738672 PMC3125189

[197] Wang Z Liu J Han J Zhang T Li S Hou Y. Herpes simplex virus 1 accelerates the progression of Alzheimer’s disease by modulating microglial phagocytosis and activating NLRP3 pathway. J Neuroinflammation. (2024) 21:1–24. doi: 10.1186/S12974-024-03166-9/FIGURES/11 39026249 PMC11264637

[198] Wu Z Zhang X Huang Z Ma K. SARS-coV-2 proteins interact with alpha synuclein and induce lewy body-like pathology. In Vitro. Int J Mol Sci. (2022) 23(6):3394. doi: 10.3390/IJMS23063394 35328814 PMC8949667

[199] Semerdzhiev SA Fakhree MAA Segers-Nolten I Blum C Claessens MMAE. Interactions between SARS-coV-2 N-protein and α-synuclein accelerate amyloid formation. ACS Chem Neurosci. (2022) 13:143–50. doi: 10.1021/ACSCHEMNEURO.1C00666 PMC8739828 34860005

[200] Idrees D Kumar V. SARS-CoV-2 spike protein interactions with amyloidogenic proteins: Potential clues to neurodegeneration. Biochem Biophys Res Commun. (2021) 554:94–8. doi: 10.1016/J.BBRC.2021.03.100 PMC7988450 33789211

[201] Jarrahi A Ahluwalia M Khodadadi H Da Silva Lopes Salles E Kolhe R Hess DC. Neurological consequences of COVID-19: what have we learned and where do we go from here? J Neuroinflammation. (2020) 17:1–12. doi: 10.1186/S12974-020-01957-4 PMC7525232 32998763

[202] Romeo MA Faggioni A Cirone M. Could autophagy dysregulation link neurotropic viruses to Alzheimer’s disease? Neural Regener Res. (2019) 14:1503–6. doi: 10.4103/1673-5374.253508 PMC6557098 31089040

[203] Panda C Mahapatra RK. Bi-directional relationship between autophagy and inflammasomes in neurodegenerative disorders. Cell Mol Neurobiol. (2023) 43:115–37. doi: 10.1007/S10571-021-01184-2 PMC11415217 35066716

[204] Lizama BN Chu CT. Neuronal autophagy and mitophagy in Parkinson’s disease. Mol Aspects Med. (2021) 82:100972. doi: 10.1016/J.MAM.2021.100972 34130867 PMC8665948

[205] Ke PY. Regulation of autophagosome–lysosome fusion by human viral infections. Pathogens. (2024) 13:266. doi: 10.3390/PATHOGENS13030266 38535609 PMC10974352

[206] Wozniak MA Frost AL Preston CM Itzhaki RF. Antivirals reduce the formation of key Alzheimer’s disease molecules in cell cultures acutely infected with herpes simplex virus type 1. PloS One. (2011) 6(10):e25152. doi: 10.1371/JOURNAL.PONE.0025152 22003387 PMC3189195

[207] Aliyu S. Viral, fungal, protozoal and helminthic infections. In: Clinical pharmacology. Churchill Livingstone (2012). p. 213–39. doi: 10.1016/B978-0-7020-4084-9.00054-9

[208] Brok J Gluud LL Gluud C. Ribavirin monotherapy for chronic hepatitis C infection: a Cochrane Hepato-Biliary Group systematic review and meta-analysis of randomized trials. Am J Gastroenterol. (2006) 101:842–7. doi: 10.1111/J.1572-0241.2006.00505.X 16494584

[209] Pancheva SN. Potentiating effect of ribavirin on the anti-herpes activity of acyclovir. Antiviral Res. (1991) 16:151–61. doi: 10.1016/0166-3542(91)90021-I 1665959

[210] Smee DF Evans WJ Nicolaou KC Tarbet EB Day CW. Susceptibilities of enterovirus D68, enterovirus 71, and rhinovirus 87 strains to various antiviral compounds. Antiviral Res. (2016) 131:61. doi: 10.1016/J.ANTIVIRAL.2016.04.003 27063860 PMC5100981

[211] Itzhaki RF Wozniak MA. Could Antivirals be used to Treat Alzheimer‘s Disease? Future Microbiol. (2012) 7:307–9. doi: 10.2217/FMB.12.10 22393884

[212] Devanand DP Andrews H Kreisl WC Razlighi Q Gershon A Stern Y. Antiviral therapy: Valacyclovir Treatment of Alzheimer’s Disease (VALAD) Trial: protocol for a randomised, double-blind,placebo-controlled, treatment trial. BMJ Open. (2020) 10(2):e032112. doi: 10.1136/BMJOPEN-2019-032112 PMC7045215 32034019

[213] Iqbal UH Zeng E Pasinetti GM. The use of antimicrobial and antiviral drugs in alzheimer’s disease. Int J Mol Sci. (2020) 21:1–19. doi: 10.3390/IJMS21144920 PMC7404195 32664669

[214] Hui Z Zhijun Y Yushan Y Liping C Yiying Z Difan Z. The combination of acyclovir and dexamethasone protects against Alzheimer’s disease-related cognitive impairments in mice. Psychopharmacol (Berl). (2020) 237:1851–60. doi: 10.1007/S00213-020-05503-1 32221697

[215] Su TH Yang HC Tseng TC Chou SW Lin CH Liu CH. Antiviral therapy in patients with chronic hepatitis C is associated with a reduced risk of parkinsonism. Movement Disord. (2019) 34:1882–90. doi: 10.1002/MDS.27848 31505068

[216] Lin WY Lin MS Weng YH Yeh TH Lin YS Fong PY. Association of antiviral therapy with risk of parkinson disease in patients with chronic hepatitis C virus infection. JAMA Neurol. (2019) 76:1019–27. doi: 10.1001/JAMANEUROL.2019.1368 PMC6551582 31168563

[217] Festa BP Siddiqi FH Jimenez-Sanchez M Won H Rob M Djajadikerta A. Microglial-to-neuronal CCR5 signaling regulates autophagy in neurodegeneration. Neuron. (2023) 111:2021–2037.e12. doi: 10.1016/J.NEURON.2023.04.006 37105172

[218] Kadowaki T Komagamine T Suzuki K Hirata K. Oseltamivir-induced dyskinesia in Parkinson’s disease. Parkinsonism Relat Disord. (2011) 17:133–4. doi: 10.1016/J.PARKRELDIS.2010.10.013 21084212

[219] Garcia-Montojo M Fathi S Norato G Smith BR Rowe DB Kiernan MC. Inhibition of HERV-K (HML-2) in amyotrophic lateral sclerosis patients on antiretroviral therapy. J Neurol Sci. (2021) 423:117358. doi: 10.1016/J.JNS.2021.117358 33653604 PMC8009857

[220] Record History | ver. 1: 2016-08-11 | NCT02868580 . ClinicalTrials.gov. Available online at: https://clinicaltrials.gov/study/NCT02868580?tab=history&a=1 (Accessed September 13, 2024).

[221] Miki K Nagai T Suzuki K Tsujimura R Koyama K Kinoshita K. Anti-influenza virus activity of biflavonoids. Bioorg Med Chem Lett. (2007) 17:772–5. doi: 10.1016/J.BMCL.2006.10.075 17110111

[222] Tatlı Çankaya İ Devkota HP Zengin G Šamec D. Neuroprotective potential of biflavone ginkgetin: A review. Life. (2023) 13(2):562. doi: 10.3390/LIFE13020562 36836918 PMC9964866

[223] Melrose J Smith MM. Natural and semi-synthetic flavonoid anti-SARS-coV-2 agents for the treatment of long COVID-19 disease and neurodegenerative disorders of cognitive decline. Front Bioscience - Elite. (2022) 14. doi: 10.31083/J.FBE1404027/PDF 36575843

[224] Wang Z Wang Y Pasangulapati JP Stover KR Liu X Schier S. Design, synthesis, and biological evaluation of furosemide analogs as therapeutics for the proteopathy and immunopathy of Alzheimer’s disease. Eur J Med Chem. (2021) 222:113565. doi: 10.1016/J.EJMECH.2021.113565 34118718

[225] Tada T Kumada T Okushin H Tani J Takaguchi K Tsutsui A. Real-world virological efficacy and safety of ledipasvir and sofosbuvir in patients with chronic hepatitis C virus genotype 2 infection: A multicenter study. Infect Dis Ther. (2021) 10:269. doi: 10.1007/S40121-020-00364-9 33141401 PMC7954884

[226] Jonker I Doorduin J Knegtering H Van’T Hag E Dierckx RA De Vries EFJ. Antiviral treatment in schizophrenia: a randomized pilot PET study on the effects of valaciclovir on neuroinflammation. Psychol Med. (2023) 53:7087. doi: 10.1017/S0033291723000430 37016791 PMC10719624

[227] Chen W Yao S Wan J Tian Y Huang L Wang S. BBB-crossing adeno-associated virus vector: An excellent gene delivery tool for CNS disease treatment. J Controlled Release. (2021) 333:129–38. doi: 10.1016/J.JCONREL.2021.03.029 33775685

[228] Zhu D Schieferecke AJ Lopez PA Schaffer DV. Adeno-associated virus vector for central nervous system gene therapy. Trends Mol Med. (2021) 27:524–37. doi: 10.1016/J.MOLMED.2021.03.010 33895085

[229] Christine CW Starr PA Larson PS Eberling JL Jagust WJ Hawkins RA. Safety and tolerability of putaminal AADC gene therapy for Parkinson disease. Neurology. (2009) 73:1662–9. doi: 10.1212/WNL.0B013E3181C29356 PMC2839805 19828868

[230] Stoker TB Torsney KM Barker RA. Emerging treatment approaches for Parkinson’s disease. Front Neurosci. (2018) 12:693/BIBTEX. doi: 10.3389/FNINS.2018.00693/BIBTEX 30349448 PMC6186796

[231] Eberling JL Jagust WJ Christine CW Starr P Larson P Bankiewicz KS. Results from a phase I safety trial of hAADC gene therapy for Parkinson disease. Neurology. (2008) 70:1980–3. doi: 10.1212/01.WNL.0000312381.29287.FF 18401019

[232] Morabito G Giannelli SG Ordazzo G Bido S Castoldi V Indrigo M. AAV-PHP.B-mediated global-scale expression in the mouse nervous system enables GBA1 gene therapy for wide protection from synucleinopathy. Mol Ther. (2017) 25:2727–42. doi: 10.1016/J.YMTHE.2017.08.004 PMC5768559 28882452

[233] Rafii M Baumann T Bakay R Ostrove J. A phase1 study of stereotactic gene delivery of AAV2-NGF for Alzheimer’s disease. Alzheimers Dement. (2014) 10(5):571–81. doi: 10.1016/j.jalz.2013.09.004 24411134

[234] Rafii M Tuszynski M Thomas R. Adeno-associated viral vector (serotype 2)–nerve growth factor for patients with alzheimer disease: a randomized clinical trial. JAMA Neurol. (2018) 75(7):834–41. doi: 10.1001/jamaneurol.2018.0233 PMC5885277 29582053

[235] Summerford C Samulski RJ. Membrane-associated heparan sulfate proteoglycan is a receptor for adeno-associated virus type 2 virions. J Virol. (1998) 72:1438–45. doi: 10.1128/JVI.72.2.1438-1445.1998 PMC124624 9445046

